# In-depth Site-specific Analysis of N-glycoproteome in Human Cerebrospinal Fluid and Glycosylation Landscape Changes in Alzheimer's Disease

**DOI:** 10.1016/j.mcpro.2021.100081

**Published:** 2021-04-20

**Authors:** Zhengwei Chen, Qinying Yu, Qing Yu, Jillian Johnson, Richard Shipman, Xiaofang Zhong, Junfeng Huang, Sanjay Asthana, Cynthia Carlsson, Ozioma Okonkwo, Lingjun Li

**Affiliations:** 1 Department of Chemistry, University of Wisconsin, Madison, Wisconsin, USA; 2 School of Pharmacy, University of Wisconsin, Madison, Wisconsin, USA; 3 Department of Applied Science, University of Wisconsin-Stout, Menomonie, Wisconsin, USA; 4 School of Medicine and Public Health, University of Wisconsin, Madison, Wisconsin, USA

**Keywords:** site-specific intact glycopeptide characterization, N-glycoproteome analysis, cerebrospinal fluid, glycopeptide enrichment, electron-transfer higher-energy collision induced dissociation (EThcD), ABC, ammonium bicarbonate, AD, Alzheimer's disease, ADRC, Alzheimer’s Disease Research Center, APP, amyloid precursor protein, ConA, concanavalin A, CNS, central nervous system, CSF, cerebrospinal fluid, DMSO, dimethyl sulfoxide, DTT, dithiothreitol, EThcD, electron transfer and higher-energy collision dissociation, FDR, false discovery rate, HILIC, hydrophilic interaction chromatography, IAA, iodoacetamide, PBA, phenylboronic acid, PTM, posttranslational modification, RCA, *Ricinus communis* agglutinin, SDS, sodium dodecyl sulfate, TFA, trifluoroacetic acid, WGA, wheat germ agglutinin

## Abstract

As the body fluid that directly interchanges with the extracellular fluid of the central nervous system (CNS), cerebrospinal fluid (CSF) serves as a rich source for CNS-related disease biomarker discovery. Extensive proteome profiling has been conducted for CSF, but studies aimed at unraveling site-specific CSF N-glycoproteome are lacking. Initial efforts into site-specific N-glycoproteomics study in CSF yield limited coverage, hindering further experimental design of glycosylation-based disease biomarker discovery in CSF. In the present study, we have developed an N-glycoproteomic approach that combines enhanced N-glycopeptide sequential enrichment by hydrophilic interaction chromatography (HILIC) and boronic acid enrichment with electron transfer and higher-energy collision dissociation (EThcD) for large-scale intact N-glycopeptide analysis. The application of the developed approach to the analyses of human CSF samples enabled identifications of a total of 2893 intact N-glycopeptides from 511 N-glycosites and 285 N-glycoproteins. To our knowledge, this is the largest site-specific N-glycoproteome dataset reported for CSF to date. Such dataset provides molecular basis for a better understanding of the structure–function relationships of glycoproteins and their roles in CNS-related physiological and pathological processes. As accumulating evidence suggests that defects in glycosylation are involved in Alzheimer's disease (AD) pathogenesis, in the present study, a comparative in-depth N-glycoproteomic analysis was conducted for CSF samples from healthy control and AD patients, which yielded a comparable N-glycoproteome coverage but a distinct expression pattern for different categories of glycoforms, such as decreased fucosylation in AD CSF samples. Altered glycosylation patterns were detected for a number of N-glycoproteins including alpha-1-antichymotrypsin, ephrin-A3 and carnosinase CN1 etc., which serve as potentially interesting targets for further glycosylation-based AD study and may eventually lead to molecular elucidation of the role of glycosylation in AD progression.

## Highlights

• Efficient N-glycopeptide sequential enrichment by HILIC and boronic acid enrichment.
• Site-specific intact N-glycopeptide characterization using EThcD.
• In-depth site-specific N-glycoproteome analysis in human CSF.
• Mapping the landscape of glycosylation patterns in Alzheimer's disease.

## Graphical abstract

**Figure:**
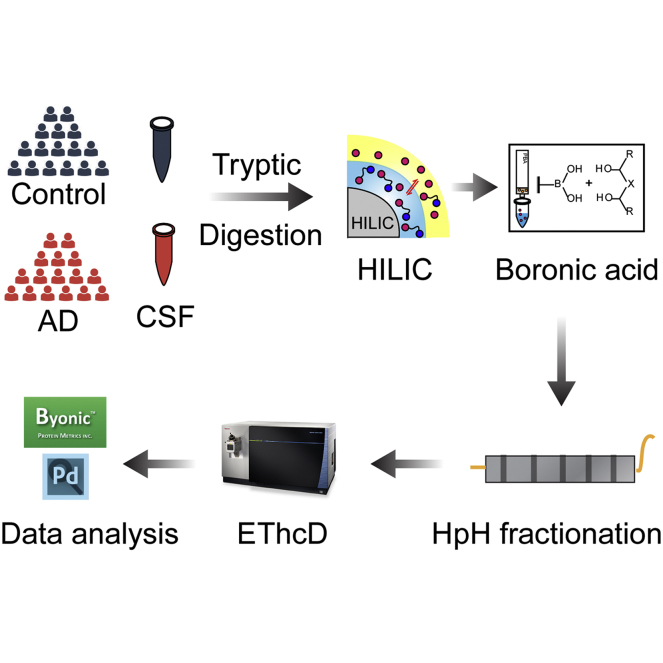

## In Brief

An exploratory glycosylation-based biomarker study has been conducted for in-depth mapping of an overall glycosylation landscape and site-specific alteration in glycoproteome collected from cerebrospinal fluids (CSF) in healthy control and Alzheimer’s disease (AD) subjects. The comparison will shed light on the glycoproteome profile, dominant glycosylation differences and similarities, and some of the interesting glycoprotein candidates with specific glycosylation pattern alterations in AD.

Originating from the brain ventricles and subarachnoid spaces around the brain and spinal cord, cerebrospinal fluid (CSF) surrounds and supports the central nervous system (CNS) (1). It is predominantly secreted by the choroid plexuses, with a minor portion produced from cerebral interstitial fluid and cerebral capillaries (2). For healthy individuals, around 80% of the CSF proteins are derived from plasma filtrate, while the remaining 20% originates directly from CNS (3). Apart from acting as a protection against mechanical trauma, CSF plays an important role in brain development, regulation of brain interstitial fluid homeostasis, and neuronal functioning. It is the only body fluid that directly interchanges with the extracellular fluid of CNS and reflects the ongoing pathological changes in the CNS most directly (4, 5). Thus, biochemical analysis of CSF has great potential for CNS-related disease diagnostics, including biomarker discovery in many neurological disorders (6, 7).

The global CSF proteome has been extensively mapped, which has largely contributed to our understanding of CNS functioning under both healthy and pathological conditions (8). Various protein posttranslational modifications (PTMs) such as phosphorylation, glycosylation, methylation etc., greatly increase the proteome complexity and functional diversity, and analysis of these “subproteomes” would further improve our understanding of protein function. However, the characterization of CSF “subproteomes” is still lagging behind proteome study due to underdeveloped and less effective analytical technologies. Among various PTMs, glycosylation represents one of the most common and complex PTMs and acts as a key regulatory mechanism controlling protein folding, cell adhesion, molecular trafficking and protein clearance, receptor activation, signal transduction, and endocytosis (9).

Defects in glycosylation in humans and their links to disease suggest that glycosylation contains a remarkable amount of biological information that can potentially help elucidate various disease mechanisms and provide potential targets for disease diagnosis and therapeutic strategies (10). In fact, glycosylation-based biomarker discovery has achieved great success for cancer research, contributing to improved cancer diagnosis and monitoring of malignant progression and prognosis (10, 11, 12, 13, 14). On the other hand, alterations in protein glycosylation have also been related to human neurodegenerative disease (ND) states, such as Alzheimer’s disease (AD), Parkinson’s disease, and Creutzfeldt–Jakob disease (15, 16). Abnormal glycosylation patterns of amyloid precursor protein (APP), microtubule-associated protein tau (tau), and numerous other proteins have been reported in AD (16). It has also been shown that O-glycosylation protects tau against aberrant phosphorylation and subsequent aggregation (17, 18, 19).

Nevertheless, there are few reports on the CSF N-glycoproteome study. One study has reported 846 N-glycosites from 520 N-glycoproteins in CSF after removing glycan part with enzyme PNGase F (8). Another study utilizing glycomics approach identified 90 N-glycan structures in human CSF (20). These studies are either protein-targeted (deglycoproteomics) or glycan-targeted (glycomics), losing the site-specific information of each individual glycan. Initial efforts into site-specific analysis of intact glycopeptides in CSF have been made, but the depth was rather limited, with one study showing the identification of 36 N-glycosites from 23 N-glycoproteins (21) and another study showing the identification of 55 N-glycosites from 36 N-glycoproteins (22). With such limited site-specific N-glycoproteome information, the process of uncovering different glycoproteins’ roles in CNS is hampered, and it will also hinder the design of studies to explore disease-related glycosylation alterations.

In the present study, we have developed an enhanced integrated large-scale site-specific glycoproteomic approach for in-depth CSF N-glycoproteome analysis, including sequential hydrophilic interaction liquid chromatography (HILIC) and boronic acid enrichment for improved N-glycopeptide coverage, intact N-glycopeptide characterization enabled by electron-transfer and higher-energy collision dissociation (EThcD), and automated false discovery rate (FDR)-based large-scale data analysis by Byonic. This approach allows us to analyze thousands of intact N-glycopeptides from CSF in a high-throughput manner, generating information about glycopeptide sequences, glycosylation site, and glycan composition. In total, 2893 intact N-glycopeptides from 511 N-glycosites and 285 N-glycoproteins were identified in CSF, representing the largest reported site-specific CSF N-glycoproteome dataset so far. This developed strategy was also applied to N-glycoproteome analysis of CSF samples from AD patients, allowing us to conduct a glycosylation pattern comparison between healthy control and AD. A comparable N-glycoproteome coverage was obtained for CSF samples collected from AD patients, but diverse and distinct glycosylation patterns were detected for glycoproteins such as alpha-1-antichymotrypsin, ephrin-A3 and carnosinase CN1 etc., which serve as promising glycosylation-based biomarker candidates for AD. This work lays a foundation for more in-depth investigation of the functional roles of these glycosylated proteins in AD progression.

## Experimental Procedures

### Chemicals and Materials

Dithiothreitol (DTT), sequencing grade trypsin were purchased from Promega. Concanavalin A (ConA), wheat germ agglutinin (WGA), *Ricinus communis* agglutinin (RCA120), iodoacetamide (IAA), acetyl-D18 glucosamine, D-lactose, methyl α-D-mannopyranoside, and manganese dichloride were obtained from Sigma-Aldrich. Tris base, urea (UA), sodium chloride, ammonium bicarbonate (ABC), calcium chloride (CaCl_2_), and ACS-grade and Optima LC/MS-grade solvents were obtained from Fisher Scientific. Formic acid (FA), 10% sodium dodecyl sulfate solution (SDS), trifluoroacetic acid (TFA), and dimethyl sulfoxide (DMSO) were purchased from Sigma-Aldrich. C18 OMIX tips and Phenylboronic acid (PBA) solid-phase extraction cartridges were obtained from Agilent. Hydrophilic interaction chromatography material (PolyHYDROXYETHYL A) was obtained from PolyLC. Microcon filters YM-30 (30 kDa) and amicon Ultra-0.5 ml centrifugal filters (10 kDa) were purchased from Merck Millipore. PANC-1 pancreatic ductal adenocarcinoma cells were from ATCC.

### CSF Samples

All study procedures involving human subjects have been approved by the University of Wisconsin Institutional Review Board and abide by the Declaration of Helsinki principles. Each enrollee was provided a signed informed consent form before participation. Thirty-two enrollees in the Wisconsin Alzheimer’s Disease Research Center (ADRC) participated in this study. The subjects comprised of 16 cognitively normal individuals who enrolled in the Wisconsin ADRC at late middle age and 16 individuals with AD dementia. Detailed subjects’ information can be found in Supplemental Table S1. All AD participants were diagnosed *via* applicable clinical criteria in standardized and multidisciplinary consensus conferences (23, 24). Cognitive normalcy was determined based on intact cognitive performance by a comprehensive battery of neuropsychological tests, lack of functional impairment, and absence of neurological or psychiatric conditions that might impair cognition (25, 26). CSF sample was collected by lumbar puncture of individuals under written informed consent. CSF aliquots from each of the 16 individuals at each stage were combined into a pool of 1 ml for control and AD subjects.

### PANC1 Cells

The proteins extracted from PANC1 cells were intended for the optimization of glycoprotein enrichment method. Commercially available PANC1 pancreatic ductal adenocarcinoma cells were routinely maintained in complete media of DMEM/Ham’s F-12 (1:1) (ATCC) supplemented with 10% fetal bovine serum (Hyclone) and 1% antibiotic-antimycotic solution (Cellgro). Cell culture flasks were placed in an incubator containing 5% CO_2_ and 98% humidity. Cells were used for a maximum of 15 passages and trypsinized using 0.25% trypsin EDTA solution (Gibco) once 80% confluence was achieved. Cell pellets were rapidly washed twice with phosphate-buffered saline, flash frozen in dry ice, and stored at –80 °C.

### Protein Extraction and Digestion From PANC1 Cells

PANC1 cell pellets were lysed by sonication in a solution containing digest buffer (4% SDS, 100 mM Tris base, pH 8). The bicinchoninic acid assay (BCA assay) was applied to determine the protein concentration. Trypsin digestion was performed based on previously reported filter-aided sample preparation protocol with some modifications (27). Briefly, proteins were thawed and centrifuged at 16,000*g* for 5 min. Then 200 μg of protein was taken out to the vial, and 1 M DTT (in digestion buffer) was added to a final concentration of 0.1 M. The sample was incubated at 95 °C for 3 min to reduce disulfide bonds. The mixture was loaded onto the 30 kDa filter and buffer exchanged with 200 μl of UA buffer (8 M UA in 100 mM Tris base) by centrifugation at 14,000*g* for two cycles (15 min each). Then sample was incubated with 100 μl of IAA (0.05 M IAA in UA buffer) in darkness for 20 min, washed with 100 μl of UA buffer for three cycles, and 100 μl of ABC buffer (50 mM) for three cycles by centrifugation at 14,000*g* (15 min each). All centrifugation was conducted at 20 °C. In total 10 μl of trypsin and 40 μl of ABC buffer were added onto the filter and the mixture was incubated at 37 °C water bath for 18 h. After incubation, the filter was transferred to a fresh collection vial and washed with 50 μl 0.5 M NaCl solution for two cycles by centrifugation at 14,000*g* (10 min each). TFA was added into the vial to a final concentration of 0.25%. Samples were then desalted using a SepPak C18 SPE cartridge following manufacturer’ protocol. Briefly, SepPak cartridge was conditioned and equilibrated with ACN and 0.1% TFA in water before loading samples. After washing with 0.1% TFA in water, peptides were sequentially eluted with 50% ACN, 0.1% FA in water, 70% ACN, 0.1% FA in water, and dried under vacuum.

### CSF Sample Processing

CSF samples were separated into peptides fraction and protein fraction using 10 kDa MWCO following the previous protocol (23, 28). The peptide fractions were analyzed in a separate study. Protein fractions were dissolved in 8 M urea/50 mM Tris HCl (pH 8), reduced with 5 mM DTT for 1 h, alkylated with 15 mM IAA for 30 min before quenching in DTT at room temperature. Protein samples were then diluted with 50 mM Tris HCl until reaching a urea concentration of <1 M before adding trypsin in a 50:1 (protein:enzyme) ratio and incubated for 18 h at 37 °C. The digestion was quenched by the addition of TFA to a final concentration of 0.3%. Finally, the samples were desalted on a C18 SepPak cartridge (Waters) and dried under vacuum.

### HILIC Enrichment

HILIC enrichment was conducted following a previously reported protocol with minor modifications (24). Namely, 5 mg of HILIC beads (PolyLC) was first activated in 100 μl elution buffer (0.1% TFA in water) by vortexing for 30 min. Then the activated beads were washed with 100 μl of binding buffer (0.1% TFA, 19.9% H_2_O, 80% ACN) for two cycles. In total, 100 μg of tryptic peptides was dissolved in 250 μl of binding buffer and mixed with beads at a 1:50 (peptide:beads) ratio. The mixture was vortexed for 1 h for N-glycopeptide binding and washed with 50 μl of binding buffer for six cycles. Flow-through was collected for sequential lectin affinity enrichment or boronic acid enrichment. N-glycopeptides were eluted by washing the beads with elution buffer (0.1% TFA in water) for five cycles and dried down under vacuum. All separation between beads and supernatant was achieved by centrifugation. The volume of binding and elution buffer were scaled up according to starting amounts of peptides.

### Lectin Affinity Enrichment

Lectin affinity enrichment was performed based on a previously reported protocol with some modifications (25, 26, 29, 30). Briefly, 200 μg of tryptic peptides was dissolved in 80 μl of 1× binding solution (1 mM CaCl_2_, 1 mM MnCl_2_, 0.5 M NaCl in 20 mM Tris base, pH 7.3), transferred to the 30 kDa filter, and incubated with 36 μl of lectin mixtures (90 μg ConA, 90 μg WGA and 90 μg RCA120 in 2× binding buffer) at room temperature for 1 h. The filter was centrifuged to remove unbound peptides at 14,000*g* for 10 min followed by washing with 200 μl of binding solution for four cycles at 18 °C. After transferring into a new collection vial, the filter was incubated with 100 μl of sugar mixtures (300 mM N-acetyl-D-glucosamine, D-lactose, methyl α-D-mannopyranoside in 1× binding buffer) at room temperature for two cycles (30 min each). N-glycopeptides were eluted by centrifugation, acidified with TFA, desalted by C18 OMIX tip, and dried down under vacuum.

### Boronic Acid Enrichment

Boronic acid enrichment was performed based on previous reported protocol with slight modifications (31). PBA cartridges were first washed with 1 ml of anhydrous DMSO for three cycles. Tryptic peptides were dissolved in 35 μl of DMSO, loaded onto the cartridge, and incubated at 37 °C for 2 h with both ends of the cartridge sealed. The unbound peptides were removed with 1 ml of anhydrous ACN for three cycles. Then glycopeptides were eluted from the cartridge after addition of 600 μl of 0.1% TFA in water and incubation at 37 °C for two cycles (1 h each) to ensure complete elution. The enriched glycopeptides were dried down under vacuum.

### High-pH Fractionation

Enriched glycopeptides were fractionated using a C18 reverse-phase column (2.1 × 150 mm, 5 μm, 100 Å) operating at 0.3 ml/min. Samples were reconstituted in 100 μl of 10 mM ammonium formate (pH 10, HPLC mobile phase A). Mobile phase B consisted of 90% ACN, 10 mM ammonium formate (pH 10). Glycopeptides were eluted with a gradient as follows: 1% A (0–3 min), 1–35% (3–50 min), 35–60% (50–54 min), 60–70% (54–58 min), 70–100% (58–59 min). Seven fractions were collected from 4 min to 62 min. The column effluent was monitored at 280 nm with a Waters 2489 UV/Visible detector. Each fraction was dried down under vacuum.

### LC-MS/MS Analysis

Samples were dissolved in 0.1% FA and analyzed on the Orbitrap Fusion Lumos Tribrid Mass Spectrometer (Thermo Fisher Scientific) coupled to a Dionex UPLC system. A binary solvent system composed of H_2_O containing 0.1% formic acid (A) and MeCN containing 0.1% formic acid (B) was used for all analyses. Peptides were loaded and separated on a 75 μm × 15 cm homemade column packed with 1.7 μm, 150 Å, BEH C18 material obtained from a Waters UPLC column (part no. 186004661). The LC gradient for intact N-glycopeptides was set as follows, 3%–30% A (18–98 min), 30%–75% A (100–108 min), and 75%–95% A (108–118 min). The mass spectrometer was operated in data-dependent mode to automatically switch between MS and MS/MS acquisition. For intact N-glycopeptides analysis, an MS1 scan was acquired from 400 to 1800 (120,000 resolution, 4e^5^ AGC, 100 ms injection time) followed by EThcD MS/MS acquisition of the precursors with the highest charge states in an order of intensity and detection in the Orbitrap (60,000 resolution, 3e^5^ AGC, 100 ms injection time). EThcD was performed with optimized user-defined charge-dependent reaction time (2+ 50 ms; 3+ 20 ms; 4+ 20 ms; 5+ 20 ms; 6 + 9 ms; 7+ 9 ms; 8+ 9 ms) supplemented by 33% HCD activation.

### Data Analysis

All raw data files were searched against UniProt *homo sapien*s reviewed database (08.10.2016, 20, 152 sequences), using PTM-centric search engine Byonic (version 2.9.38, Protein Metrics) incorporated in Proteome Discoverer (PD 2.1). Trypsin was selected as the enzyme and two maximum missed cleavages were allowed. Searches were performed with a precursor mass tolerance of 10 ppm and a fragment mass tolerance of 0.01 Da. Static modifications consisted of carbamidomethylation of cysteine residues (+57.02146 Da). Dynamic modifications consisted of oxidation of methionine residues (+15.99492 Da), deamidation of asparagine and glutamine (+0.98402 Da), and N-glycosylation on asparagine. Oxidation and deamidation were set as “rare” modification, and N-glycosylation was set as “common” modification through Byonic node. Two rare modifications and one common modification were allowed. Human N-glycan database embedded in Byonic, which contains 182 glycan entities, was used. Results were filtered to a 1% protein FDR as set in the Byonic parameters, and data was further processed to 1% FDR at the PSM level using the 2D-FDR score (a simple variation of the standard target-decoy strategy that estimates and controls PSM and protein FDRs simultaneously) (32, 33). Only those N-glycopeptides with Byonic score >100 and |logProb| > 1 were reported (the absolute value of the log base 10 of the protein *p*-value). Each glycopeptide identified should have the consensus motif NX/T/S, X ≠ P. The filtering criteria has been reported to result in confident glycosite assignment at glycopeptide spectral match level (34).

### Experimental Design and Statistical Rationale

**Fig. 1:**
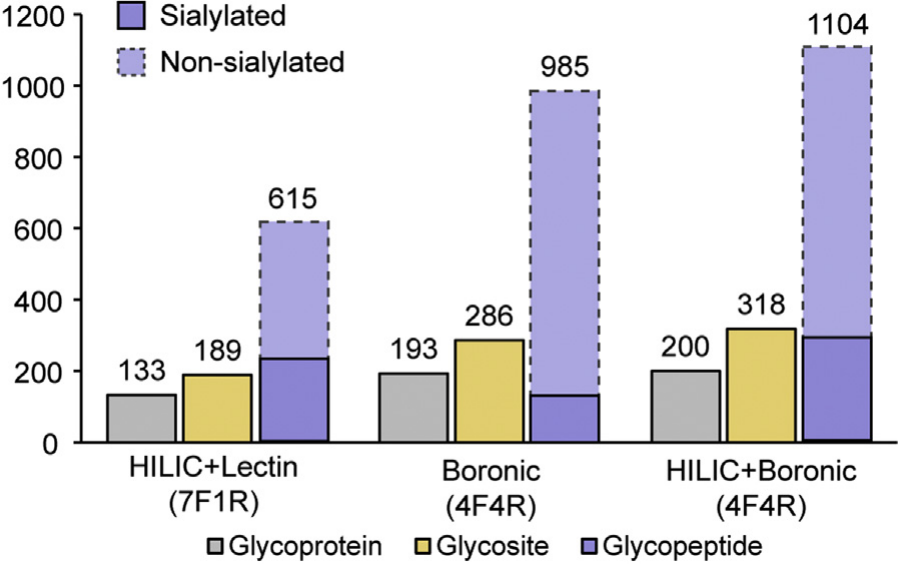
**N-glycopeptide enrichment strategy optimization.** Different enrichment strategies including either single enrichment or sequential enrichment were employed to enrich N-glycopeptides from tryptic peptides derived from 200 μg proteins extracted from PANC1 cells. The number of N-glycoproteins, N-glycosites, and N-glycopeptides of each strategy were compared. The combination of HILIC and boronic acid enrichment gives the highest coverage. (F: fractions, R: technical replicates).

Firstly, different N-glycopeptide enrichment methods and their combinations were evaluated to optimize enrichment strategy and maximize the glycoproteome coverage. Three commonly used enrichment methods including HILIC, lectin, and boronic acid were evaluated respectively, and the benefits of high pH fractionation were also investigated. For method development utilizing PANC1 cells, different number of technical replicates were conducted as shown in Figure 1 depending on the complexity of the enriched N-glycopeptides. For human CSF N-glycoproteome analysis, CSF samples from 16 healthy individuals were pooled together to obtain 1 ml sample. Trypsin digestion and following optimized enrichment strategies were applied to the CSF samples. A total of five fractions of enriched glycopeptides were collected, including one fraction from HILIC enrichment and four fractions from boronic acid enrichment. For each fraction, two technical replicates were performed during LC-MS/MS analysis. Meanwhile, the same amount of CSF samples from 16 AD patients were processed in parallel to get an N-glycoproteome landscape in AD state. As a descriptive and qualitative comparison of site-specific N-glycoproteome between healthy control and AD state was conducted, no statistical tool was used. Further discussions could be found in the Discussion section.

## Results

### Confidence of Glycopeptide Assignment

It is challenging to implement accurate and meaningful probability-based FDRs in glycoproteomics due to the additional structural complexity of glycopeptides (34). Given the limitations of the database search strategy in Byonic, it is necessary to use additional information to increase the glycopeptide assignment confidence. One approach is to measure the proteomes of the enriched, PNGase F deglycosylated, glycopeptide samples. The glycoprotein abundance from Byonic can then be correlated with deglycosylated protein abundance, and those Byonic results that correlate with the order of deglycosylated protein abundances will be considered confident. Here, we conducted proof-of-principle experiments utilizing mouse brain tissue to increase glycopeptide assignment confidence. Experimental details are provided in Supplemental data. For the glycoprotein sample and the deglycosylated sample, 372 N-glycoproteins and 1337 deglycosylated proteins were identified, respectively (Supplemental Fig. S1*a*, Supplemental Table S2). Although deglycosylated sample was derived from enriched glycoprotein sample, there were only 236 proteins shared between two cohorts. This can be explained by nonglycopeptides coeluted during enrichment process due to nonspecific binding. After PNGase F digestion, the interference of nonglycopeptide became larger and thus those low-abundance glycopeptides could not be detected in deglycosylated sample. Glycoprotein intensities were plotted against deglycosylated protein intensities in scatter plot for the 236 shared proteins (Supplemental Fig. S1*b*). Most glycoproteins correlated well with their deglycosylated protein abundance, which supports the validity of glycopeptide assignments from Byonic software under our filtering criteria.

### Optimization of Enrichment Strategies

Although advances in various analytical technologies have made large-scale analysis of glycopeptides feasible, the depth of glycoproteome study in a complex sample has been limited compared with other PTMs studies, such as phosphoproteomics. This is because glycopeptides only constitute a minor portion (2% to 5%) of the total peptide mixtures, and the signal is often suppressed in the presence of other more abundant nonglycosylated peptides (35, 36). The glycoform heterogeneity (microheterogeneity) at each glycosite will further reduce the relative abundance of each unique glycopeptide. Thus, glycopeptide enrichment is a key step in the success of glycopeptide analysis in complex biological samples. Many enrichment strategies have been developed in recent years, including HILIC, titanium dioxide (TiO_2_) affinity, lectin affinity enrichment, hydrazide, and boronic acid chemistry (11, 37, 38, 39, 40, 41, 42, 43). To further reduce sample complexity and improve glycoproteome coverage, off-line fractionation such as high-pH fractionation (HpH) is often utilized due to its ease of implementation and its compatibility with large amounts of starting material, which has also been shown highly orthogonal to the subsequent LC-MS/MS analysis with low-pH reversed-phase chromatography (44).

Boronic acid approach was first employed in this study due to its less biased enrichment mechanism. During the enrichment process, a strong reversible covalent bond will form between boronic acid and 1,2/1,3 cis-diols of any glycan moiety under alkaline conditions (45), while its reversible property allows the intact glycopeptides to be easily released under acidic conditions without any side effect. Proteins extracted from PANC1 cells were used for method optimization. With 200 μg of proteins as the starting material, boronic acid enrichment was originally combined with seven HpH fractions. However, a closer look at the number of N-glycopeptides identified in the seven HpH fractions showed that N-glycopeptides were mainly in fractions 2, 3, and 4 as shown in Supplemental Figure S2*a*, whereas the number of peptides identified were almost evenly distributed in seven fractions in a global proteomics study as shown in Supplemental Figure S2*b*. This is not surprising considering the increased hydrophilicity of glycopeptides, which results in an earlier elution on a reversed-phase C18 column. Thus, another round of enrichment combining fractions 1, 5, 6, and 7 with four technical replicates was conducted, resulting in 985 N-glycopeptide as shown in Figure 1.

HILIC and lectin affinity enrichment are other two most widely used strategies, with enrichment occurring at the intact glycopeptide level fully preserving the native glycan information. Here we compared glycopeptide enrichment performance of boronic acid, HILIC, and lectin affinity approaches as shown in Supplemental Figure S3. Boronic acid approach clearly outperformed other two methods regarding number of N-glycopeptide identifications, which further demonstrated its less biased enrichment capacity. However, HILIC enrichment showed the highest identification number of a specific species, sialylated glycopeptides, which play essential roles in many biological systems. This bias is probably because sialic acid increases the hydrophilic interactions between the glycopeptides and HILIC beads, resulting in a preferential enrichment of sialylated N-glycopeptides (46). These complementary results proved that single enrichment strategy is usually insufficient to enrich all kinds of glycopeptides due to the inherent glycopeptide structure complexity. Thus, we decided to combine multiple enrichment strategies to maximize the glycoproteome coverage of a certain sample.

With the same amount of starting material (200 μg of proteins), two sequential enrichment approaches combining HILIC with lectin, HILIC with boronic acid, followed by HpH fractionation were compared. For sequential HILIC with lectin approach, 615 intact N-glycopeptides were identified combined with seven HpH fractions (Fig. 1). Lectin affinity enrichment relies on certain glycan motif recognition, while HILIC takes advantage of increased hydrophilicity due to glycan attachment. Both methods are somewhat biased toward certain categories of glycopeptides, and these glycopeptides with lower lectin binding affinity or more hydrophobic peptide sequences may be lost. In addition, hydrophilicity overlapping region exists between glycopeptides and nonglycosylated peptides, which results in nonspecific enrichment of nonglycosylated peptides and hampers glycopeptide detection (47). On the other hand, sequential HILIC with boronic acid approach demonstrated the best performance. A total of 1104 N-glycopeptides were identified, with an increase of 119 N-glycopeptides compared with boronic acid enrichment alone (Fig. 1). Benefiting from using HILIC, the number of sialylated glycopeptides identified had a 2.2-fold increase (Fig. 1, Supplemental Table S3). Such improvement can be significant for biological samples when high percentage of glycopeptides are sialylated and thus is beneficial to capture a more complete glycosylation pattern to help understand disease mechanisms.

### Site-specific Intact N-glycopeptide Characterization

**Fig. 2:**
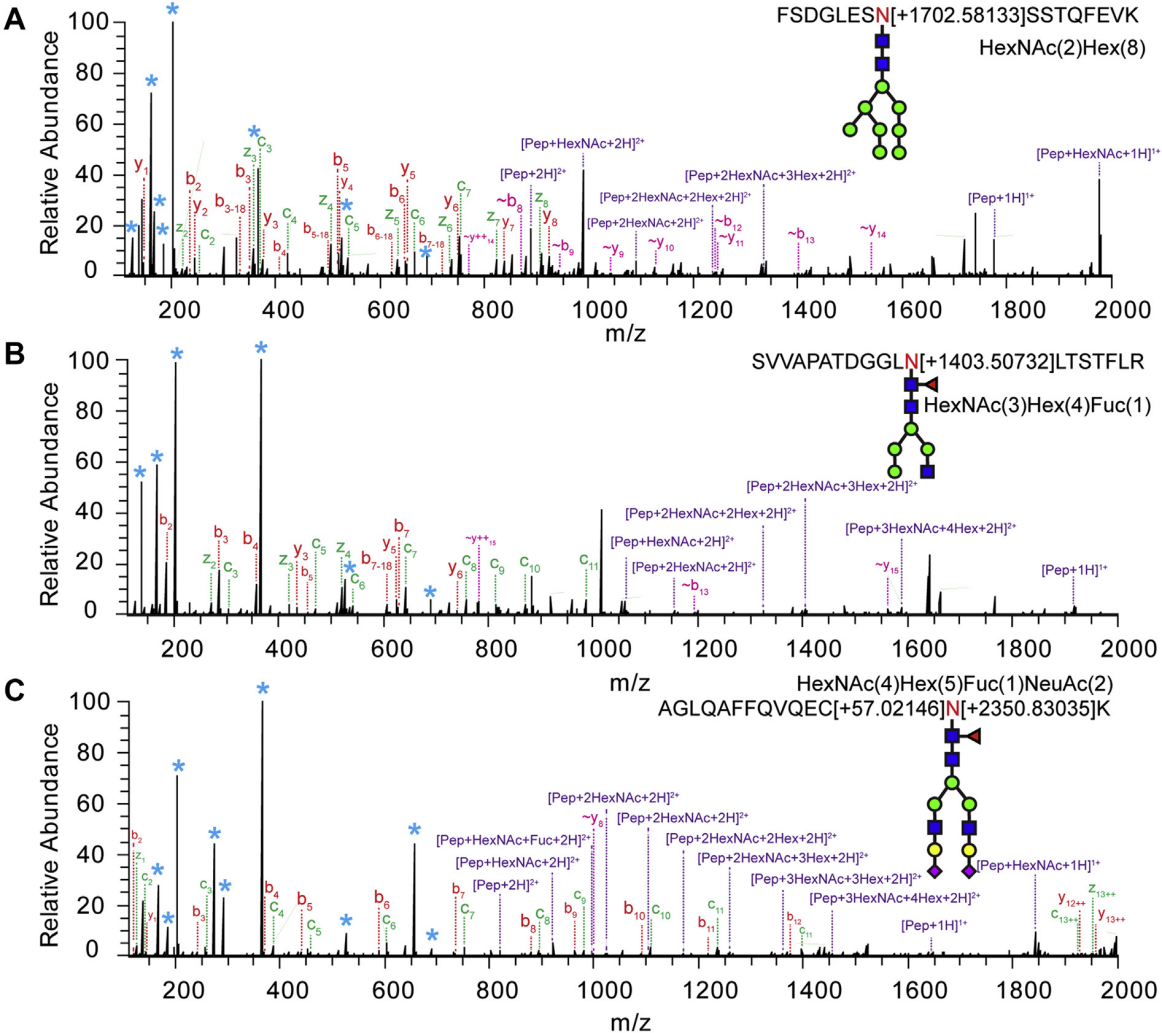
**Representative EThcD fragmentation spectra of intact N-glycopeptides with different categories of N-glycans attached are shown, including high-mannose N-glycan (*A*), hybrid N-glycan (*B*), and complex N-glycan (*C*).** Peptide fragment ions are denoted as b/y, c/z ions using the most commonly employed nomenclature, and ∼b/y ions denote the loss of labile glycan. (Asterisk: glycan oxonium ion and glycan fragment ions, “pep”: peptide sequence, HexNAc: acetylglucosamine, Hex: hexose, Fuc: fucose, NeuAc: N-acetylneuraminic acid).

Since its first introduction by Heck and coworkers (48), EThcD has shown great potential for labile PTMs analysis (*e.g.*, phosphorylation) with improved site localization, as well as generating richer backbone fragmentation spectra (49). When applied to intact glycopeptide characterization, EThcD can produce rich fragment ion information for glycan (B/Y ions), peptide (b/y, c/z ions), and glycosylation site (c/z ions) identification, providing the opportunity for site-specific intact glycopeptide analysis (50, 51). The representative EThcD fragmentation spectra, deriving from N-glycopeptides with three main N-glycan classes including high-mannose, hybrid, and complex types attached, were shown in Figure 2, *A*–*C*, respectively. A series of fragment ions including c/z, b/y ions, glycan fragment ions, and glycopeptide with one or more loss of monosaccharides were detected. The highly abundant oxonium ions were detected in the lower mass region, including 138.06 (HexNAc-2H_2_O-CH_2_O), 168.06 (HexNAc-2H_2_O), 186.08 (HexNAc-H_2_O), and 204.09 (HexNAc), confirming that the spectra were produced from a glycopeptide. For the fucosylated and sialylated glycopeptides, the signature fragment ions supporting the presence of fucose (*i.e.*, HexHexNAcFuc) and sialic acid (*i.e.*, NeuAc, NeuAc-18) were used to further confirm their identity apart from the accurate mass matching of precursor ions. As shown in Figure 2*C*, the sialic acid containing glycan fragment ion 657.23 (HexNAcHexNeuAc) along with the precursor accurate mass matching (0.5 ppm) confirms the presence of sialic acid. The intact glycopeptide ion with a HexNAc loss (peptide + HexNAcFuc, +2, 994.96) confirms the presence of fucose and indicates that the fucose group is attached to the innermost HexNAc. Peptide sequence AGLQAFFQVQECNK is deduced based on the abundant backbone fragment ions including both b/y and c/z ions. The peptide, belonging to ceruloplasmin, has an N-glycan consensus motif at 358th asparagine (Asn, N) residue, and it has been reported as an N-glycosite previously (52).

### Site-specific Glycoform Mapping in CSF

**Fig. 3:**
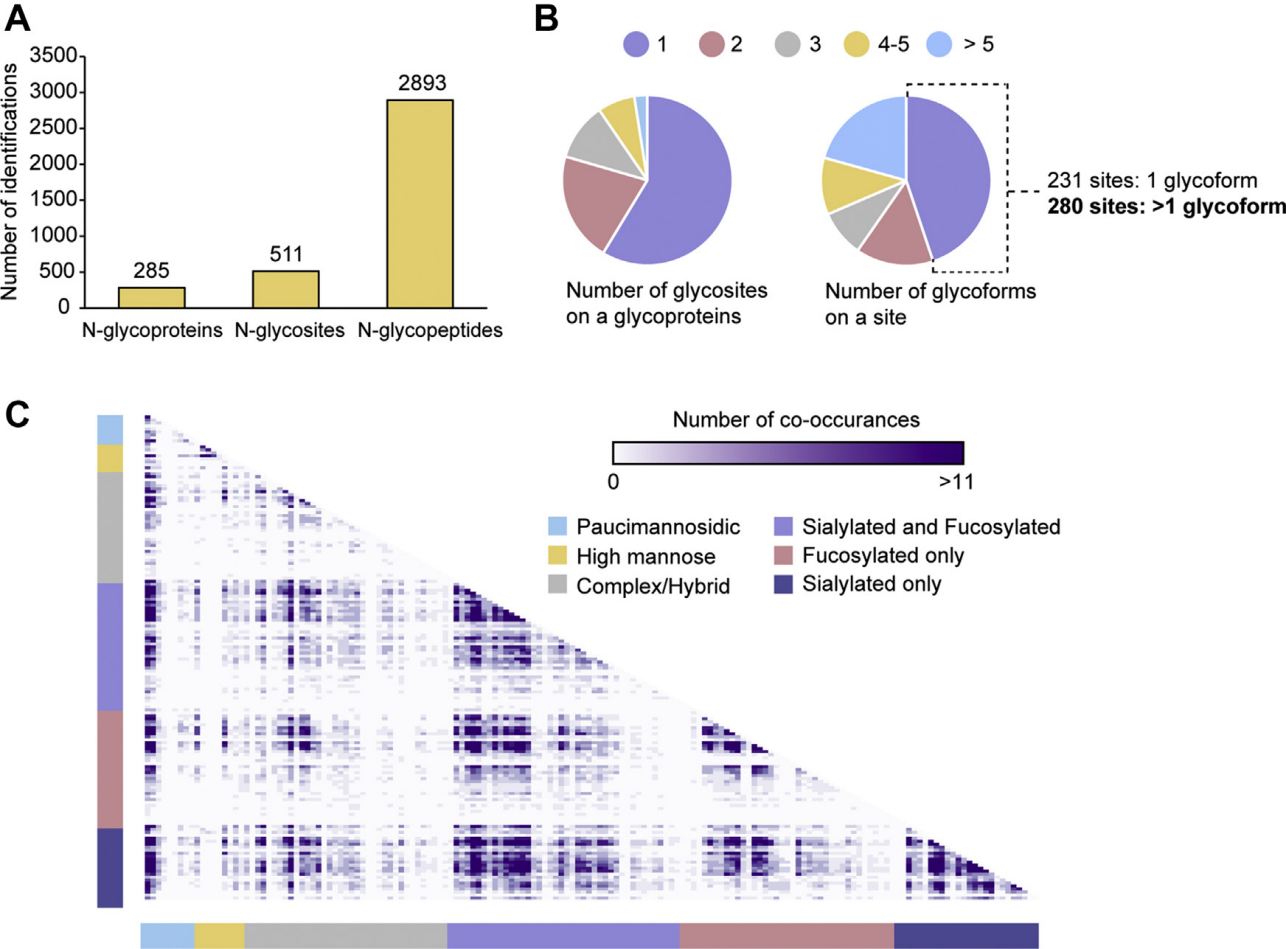
**Site-specific N-glycoproteome profiling in CSF samples from healthy control.***A*, the number of N-glycoproteins, N-glycosites, and N-glycopeptides identified. *B*, N-glycosite distribution among glycoproteins and N-glycoform distribution among glycosites. *C*, glycan co-occurrence heat map represents how many times glycan pairs co-occurred at the same site.

As discussed previously, the current depth of site-specific N-glycoproteome in CSF is far from being satisfactory, which will largely hinder glycosylation-based biomarker discovery studies in CSF for various diseases. Here, the developed enhanced glycoproteomic strategy was applied to the in-depth site-specific N-glycoproteome analysis of the CSF samples. In total, 2893 intact N-glycopeptides from 511 N-glycosites and 285 N-glycoproteins were identified (Fig. 3*A*, Supplemental Table S4), representing the largest site-specific CSF N-glycoproteome dataset so far. Majority of glycoproteins identified in this study were also detected in a previous global CSF proteomics analysis, and a positive correlation between glycoprotein and global protein abundance supports the validity of identified glycoproteins from our Byonic results (Supplemental Fig. S4, Supplemental Table S5) (53). To further improve the confidence of identified N-glycosites and N-glycoproteins, we compared the results with N-GlycositeAtlas database (54). Venn diagrams indicated the overlap of identified N-glycoproteins and N-glycosites with reported all and CSF-specific N-glycoproteome (Supplemental Fig. S5, Supplemental Table S5). Around 66% and 50% of our glycoproteins and glycosites have been reported in human CSF samples. Moreover, ∼99% and ∼92% of the glycoproteins and glycosites have been reported in previous human N-glycoproteomic studies. In addition, we compared Byonic results with other glycoproteomics search programs including pGlyco and MSFragger-Glyco (detailed search parameters in Supplemental Information) (55, 56). Venn diagrams showed the overlap of 511 glycosites identified by Byonic with other two software platforms (Supplemental Fig. S6, Supplemental Table S6). Despite different searching algorithm, large portion of our glycosites can be cross-validated by pGlyco and MSFragger-Glyco, which further increases the confidence of our glycopeptide assignments.

To obtain an overview of the cellular component, molecular functions, and biological processes of the 285 N-glycoproteins identified, Gene Ontology (GO) functional enrichment analysis was conducted using DAVID tool (http://david.abcc.ncifcrf.gov/) (57). A recently published comprehensive human CSF proteome list including 5941 proteins was used as background during the analysis (58). The majority of the identified glycoproteins are mainly distributed in extracellular region and membrane or cell surface, agreeing well with the fact that most of N-glycoproteins are membrane or secreted proteins (59). In terms of molecular functions, the top five enrichment clusters are serine-type endopeptidase inhibitor activity, heparin binding, extracellular matrix structural constituent, immunoglobulin receptor binding, and protease binding. Many biological processes that are known to involve glycosylation are enriched, including cell adhesion, negative regulation of endopeptidase activity, platelet degranulation, complement activation, and axon guidance (Supplemental Table S7).

In terms of the glycosite distribution among the glycoproteins, more than half of them (59%) carry only one glycosite, and the majority (more than 90%) of them have fewer than or equal to five glycosites (Fig. 3*B*). Only ten N-glycoproteins carry more than five glycosites, which include clusterin, galectin-3-binding protein, BDNF/NT-3 growth factors receptor, attractin, sortilin-related receptor, cell adhesion molecule 1, contactin-1, multiple epidermal growth factor-like domains protein 8, neuronal cell adhesion molecule, and IgGFc-binding protein (FcγBP) (Supplemental Table S8). The results were in accordance with previous findings that these proteins were highly N-glycosylated (60, 61). Among them, FcγBP was found to carry the largest number of N-glycosites, with ten N-glycosites detected. According to sequence analysis, it could have 33 potential N-glycosites with typical N-glycan consensus motif (NXT/S, X ≠ P), and nine N-glycosites were recorded in the Uniprot without any microheterogeneity information available (61). FcγBP is a secretory mucin-like glycoprotein present widely throughout mucous membranes and in external body fluids (62). The importance of O-glycosylation has long been recognized in the processing and biological properties of mucins, and the role of N-glycosylation starts to come to light and has been found to support mucin stability, folding, sorting, membrane trafficking, and secretion recently (63, 64). Due to a lack of glycosylation microheterogeneity information available at each site, further study into its more specific and precise biological role is hampered (65). A total of 17 N-glycoforms were identified on the ten detected N-glycosites, with four N-glycoforms on Asn5186, two N-glycoforms on Asn2138, Asn3339, Asn3719 and Asn75, and one N-glycoform on Asn1743, Asn2518, Asn2944, Asn4145, and Asn4540, respectively. With the site-specific glycoform information available, it provides an opportunity for a more in-depth investigation into the biological role of N-glycosylation on FcγBP as a whole glycoprotein.

More than half of the glycosites (60%) carried less than or equal to two glycoforms, whereas there were 20% glycosites with more than five glycoforms and 1.6% glycosites with more than 50 glycoforms (Fig. 3*B*). On average, each N-glycosite carried ∼5.1 glycoforms and each N-glycoprotein carried ∼9.2 glycoforms, suggesting a highly diverse microheterogeneity. For example, 51 glycoforms were identified at Asn93 on alpha-1-acid glycoprotein 2 (AGP-2), including 7 complex/hybrid (neither fucosylated nor sialylated), 1 high-mannose, 7 fucosylated only, 16 sialylated only, and 19 fucosylated and sialylated N-glycans. AGP-2 is an acute-phase glycoprotein containing 45% carbohydrate and was once considered to be the protein with the highest carbohydrate content (66). It mainly contains complex N-glycans and their variation (branched, sialylated, and fucosylated) has been shown to be sensitive to various pathophysiological conditions (67, 68). Various glycoforms indicate that AGP-2 has plenty of flexibility altering the N-glycosylation pattern in response to different physiological or external stimuli.

To further explore site-specific microheterogeneity, we plotted a glycan co-occurrence heat map by calculating how many times glycan pairs co-occurred at the same site (Fig. 3*C*). The glycan pair combinations (glycans that appeared together at the same glycosite) were demonstrated, and the darker color indicates more incidences of co-occurrence. We found that fucosylated and sialylated glycans appear to co-occur together with high frequency. As two of the most common and important “capping” reactions to elongate the glycan branch, sialylation and fucosylation play key roles both in biological processes such as cellular recognition, cell adhesion, cell signalingand in altered glycosylation associated with disease progression (10). In CSF, 46% and 49% glycoforms detected in our study were fucosylated and sialylated, respectively. Such a high percentage of sialylated glycopeptides detected in our study possibly resulted from the sequential enrichment using HILIC and boronic acid enrichment, especially the preferential enrichment of sialylated glycopeptides by HILIC. Furthermore, we also found co-occurrence patterns of paucimannosidic glycans with other groups of complex/hybrid, fucosylated, and sialylated glycans (Fig. 3*C*). Paucimannosylation was discovered initially in plants and invertebrates (69, 70). Growing evidence has shown that paucimannosylation is also present in mammals, including mouse embryonic neural stem cells, human buccal epithelial cells, human colorectal cancer epithelial cells, etc. (71, 72, 73, 74, 75). Although paucimannosylation in the extracellular environment is believed to be a feature of cells to communicate within the immune system and altered expression of paucimannosidic epitopes has been implicated in cancer, their exact physiological functions and roles in diseases remain largely unknown (76). The discovery of these truncated N-glycans in CSF proteins lays a foundation for future investigation of their biological roles.

### Glycosylation Alteration in AD

**Fig. 4:**
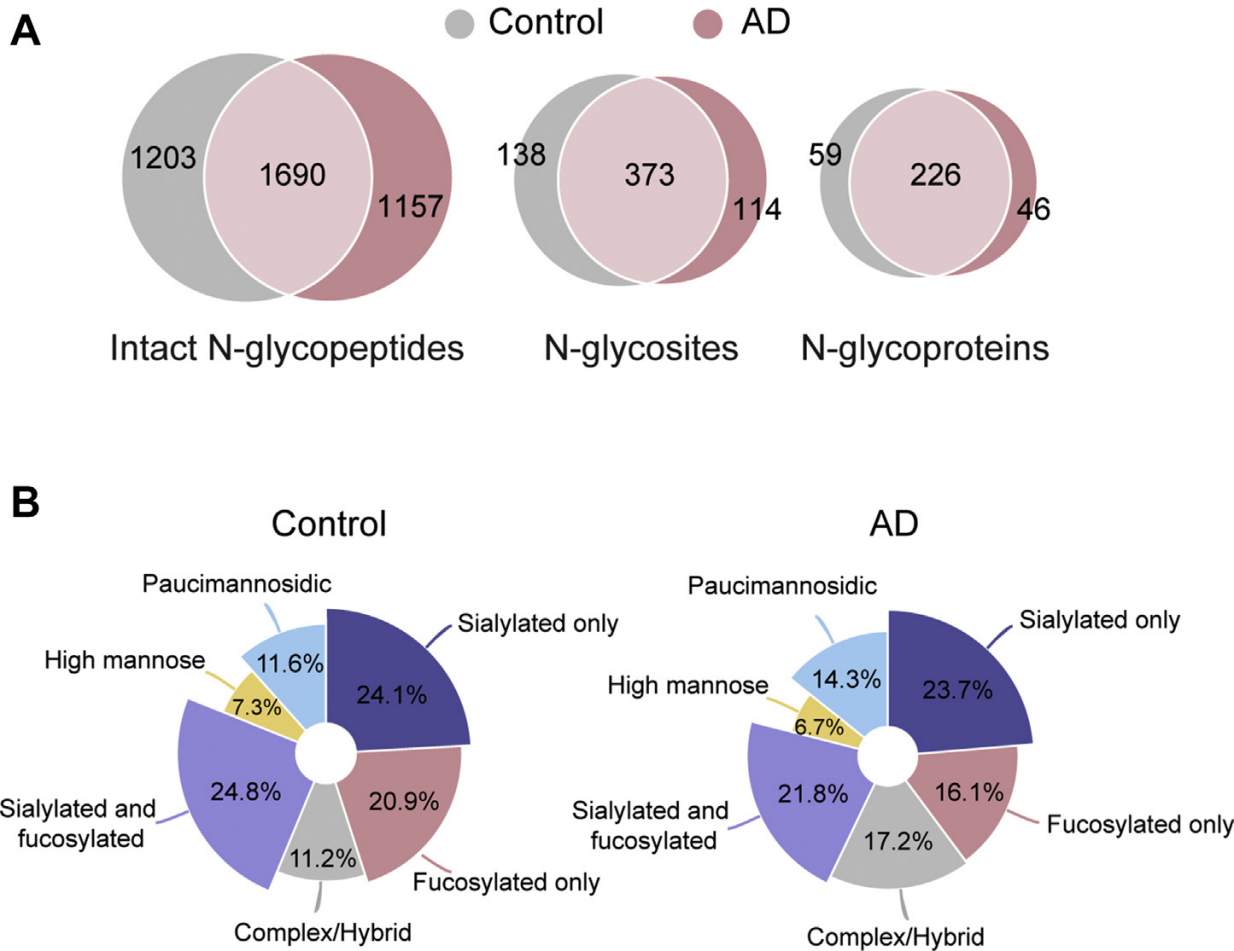
**Comparison of CSF N-glycoproteome between healthy control and AD**. *A*, Venn diagram analysis of N-glycoproteins, N-glycosites, and N-glycopeptides identified between healthy control and AD. *B*, the percentage of different N-glycoforms in CSF between healthy control and AD.

As discussed previously, numerous reports demonstrated that glycosylation alteration is implicated in the pathophysiological development of AD. To obtain a landscape of glycosylation pattern of proteome in the CSF from AD subjects, CSF samples pooled from 16 AD patients were subjected to the same in-depth N-glycoproteome analysis. To minimize the variations brought by sample preparation, the AD CSF samples were processed simultaneously with control CSF samples. In total, 2847 N-glycopeptides, 487 N-glycosites, and 272 N-glycoproteins were identified in AD CSF (Fig. 4*A*, Supplemental Table S4), which was quite comparable with the coverage in control CSF. Venn diagrams between AD and control indicate that diverse glycosylation patterns are present during AD development, with 42% overlapping for N-glycopeptides, 60% for N-glycosites, and 68% for N-glycoproteins (Fig. 4*A*). These results also suggest that an in-depth glycoproteomic study focusing on qualitative characterization of the glycoform changes is necessary before any quantitative glycoproteomic study being conducted. Based on the degree of sialylation, fucosylation, and composition complexity, these glycoforms were divided into six categories. As shown in Figure 4*B*, diverse glycoform compositions were found between healthy control and AD CSF samples.

**Fig. 5:**
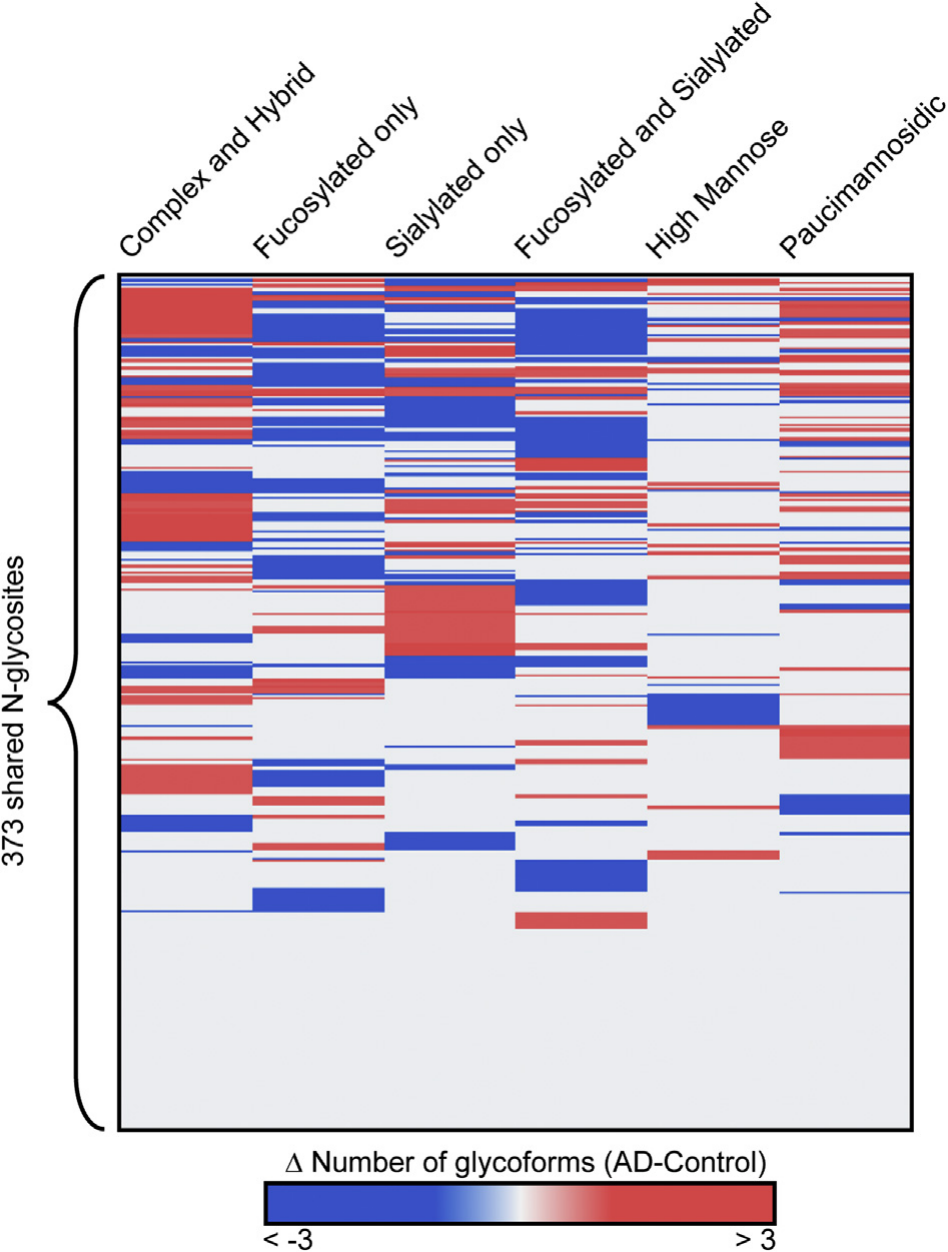
**Hierarchical clustering analysis based on delta number of detected glycoforms (changes in the number of AD-Control) visualized in heatmap**. The columns represent six categories of glycoforms, and rows represent specific N-glycosites. The delta number of glycoforms is color-coded.

For the detected 373 N-glycosites shared by control and AD, a heat map analysis was conducted to give a bird’s eye view of the glycosylation landscape alterations in AD (Fig. 5, Supplemental Table S9). Hierarchical clustering was conducted based on delta number of detected glycoforms (changes in the number of AD *versus* Control) in which blue indicates a decreased number and red indicates an increased number in AD. As shown at the bottom of the heatmap, no change of glycosylation pattern was found on around 1/3 of the shared glycosites. However, among other shared glycosites, a general trend of decreased fucosylation in AD was noted upon close examination. To get a clearer idea of the identities of these altered N-glycoproteins/N-glycosites and their glycoform changes, a two-dimensional plot depicting the changes in the number of glycoforms as a function of the number of total identified glycoforms in AD was constructed (Supplemental Fig. S7). These interesting glycoprotein candidates with glycosylation pattern changes will be discussed in a later section. Besides the shared N-glycoproteins/N-glycosites detected in both control and AD, there are 138 unique N-glycosites, 59 unique N-glycoproteins detected in control CSF sample and 114 unique N-glycosites, 46 unique N-glycoproteins in the AD CSF sample (Supplemental Tables S10 and S11). These uniquely present N-glycoproteins/N-glycosites could also be potential markers for AD as these diverse glycosylation patterns may indicate differential regulation of biological processes in disease states (77). Selected interesting potential biomarker candidates with altered glycosylation patterns are discussed below.

## Discussion

The current knowledge of site-specific protein glycoforms in CSF is quite limited due to the inherent structural complexities of intact glycopeptides and less well-developed analytical technologies compared with other PTMs studies. Benefiting from the improved workflow, including optimized sequential glycopeptide enrichment and intact N-glycopeptide characterization enabled by EThcD, thousands of intact N-glycopeptides from CSF were identified in this work. In order to map the site-specific glycoforms in human CSF, an in-depth glycoproteomic analysis was conducted using the CSF samples pooled together from 16 healthy subjects. The identified 2893 intact glycopeptides from 285 N-glycoproteins represent the largest site-specific N-glycoproteome dataset for human CSF analysis so far. As many studies have shown that the glycosylation alteration is implicated in AD progression, it would be highly valuable to obtain a description of the N-glycoproteome landscape of CSF in AD and make a comparison to that of healthy control.

Unlike in bottom-up proteomics studies, where different proteoforms contributed by PTMs are not fully characterized and quantitation is typically conducted at the protein level, glycoproteomic studies often target at each individual intact glycopeptide, which involves the characterization of various glycoforms at each site (78). Due to this reason, there are two aspects for glycosylation alterations in a glycoproteomic study, the abundance changes and the composition changes of glycoforms at the same site, and the latter includes increased/decreased sialylation, fucosylation and branching etc. (10). Therefore, for any glycoproteomic-driven biomarker discovery study, it is desirable to comprehensively characterize the glycoforms and determine the glycoform changes involved in the disease and control states before a quantitative study is conducted.

Thus, an in-depth qualitative glycoproteomics study was conducted focusing on exploring the glycoform changes in AD patients using the CSF samples pooled from 16 age-matched AD patients. Although ideally an in-depth glycoproteomic study conducted on individual control and AD subject would allow us to account for any individual variation, the limited CSF sample amount, instrument/labor time, and financial constraints were the limiting factors that made such experiments difficult to carry out (79). While pooling the samples together for in-depth glycoprotein profiling comparison between disease and control offers an effective and efficient way for initial screening of interesting disease-related biomarkers, at the same time it also increases the chance of the detection of low-abundance glycoproteins. It has been shown that pooling samples reduce biological variation and increase statistical power because pools represent averages, and the dominant differences between experimental groups might be easier to find (80). In total, a comparable number of intact N-glycopeptides were identified in AD CSF samples, but with a diverse composition of different glycoforms, indicating that AD might affect the physiology by manipulating glycosylation in a subtle and delicate way instead of massive glycoform changes. Utilizing pooled CSF samples, we were still able to identify diverse glycosylation patterns, which indicates that larger variations exist between healthy and disease states. There are a few glycoproteins/glycosites showing altered glycosylation patterns. Here, some of the interesting targets have been selected for further discussion.

**Fig. 6:**
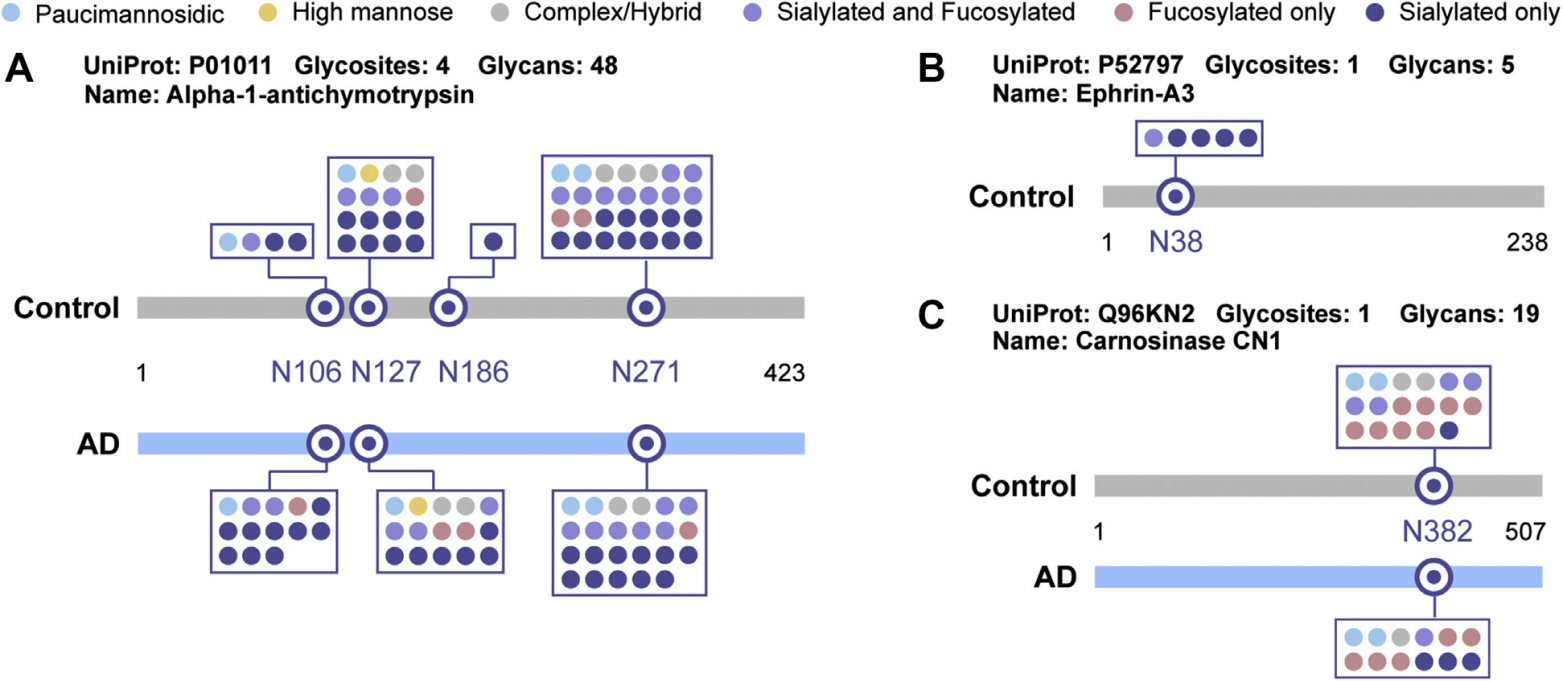
**Glycosylation microheterogeneity for alpha-1-antichymotrypsin (*A*), ephrin-A3 (*B*), and carnosinase CN1 (*C*) detected in CSF between healthy control and AD**.

As a member of the serine protease inhibitor family of acute-phase proteins, alpha-1-antichymotrypsin (ACT) is predominantly synthesized in the liver and also produced in the brain by the astrocytes (81). Studies have shown increased ACT levels in serum and CSF collected from AD patients, indicating that ACT may serve as a candidate biomarker for early AD diagnosis (82). *In vitro* experiments have demonstrated that ACT binds to Aβ peptides and promotes the assembly of the Aβ peptides into amyloid filaments (83, 84), and later *in vivo* studies using transgenic AD mouse models confirmed that ACT is an integral component of the amyloid deposits and accelerates amyloid plaque formation (85, 86). Additionally, ACT has also been shown to induce tau phosphorylation in neurons and subsequent neuronal cell apoptosis (87). ACT is an N-glycoprotein with estimated 24% of carbohydrate contents distributed among the six potential N-glycosites (88). The glycan moiety compositions have been partially revealed by previous studies of the extracted plasma ACT using affinity immuno-electrophoresis and NMR spectroscopy (89, 90), showing evidence for disialyl diantennary, trisialyl triantennary, disialylated triantennary structures. Glycosylation patterns of acute phase proteins (*i.e.*, ACT) in response to chronic inflammatory diseases (*i.e.*, AD) have been extensively studied as potential biomarkers and reviewed recently (91). In fact, altered glycosylation profiles (reduced terminal GlcNAc and sialic acid) have been detected on the purified plasma ACT in AD patients (92). However, in the previous study, lectin-assisted glycan array analysis was used, which could not provide precise information about the exact microheterogeneity alterations for each putative N-linked glycosite. No studies to date have explored the glycosylation alterations in ACT in CSF. Benefiting from the site-specific glycosylation analysis of the proposed workflow, our study elucidated the alterations of glycosylation micro-heterogeneity on each site (Fig. 6*A*). In total, 4 N-glycosites (Asn106, Asn127, Asn186, Asn271) were detected for ACT in AD patients and healthy control. The results show that there was an increase in the complex N-glycans, especially sialylated glycans, on site 106 and 186, while a comparable number of N-glycans were detected on site 127 and 271.

Another interesting glycoprotein target with altered glycosylation in AD is the membrane ligand for Eph receptors, ephrin-A3. Although ephrin-A3 has been shown to have three potential N-glycosites (Asn38, Asn67, Asn100) by Uniprot sequence analysis, there has been no experimental evidence to confirm the N-linked glycosylation at these putative glycosites so far. In accordance with the Uniprot sequence analysis, our results unambiguously identified Asn38 as the glycosite of ephrin-A3 in CSF (Fig. 6*B*). A total of five glycoforms were identified on this site in ephrin-A3 in CSF collected from healthy control, while no glycosylated ephrin-A3 was identified in AD samples. The Eph receptors belong to the superfamily of transmembrane Tyr kinase receptors, and the Eph/ephrin pathway mediates short-distance cell–cell communication after activation (91). In the body, Eph/ephrin pathway regulates various developmental processes, including cardiovascular and skeletal development, as well as axon guidance, synapse formation, maintenance, and plasticity in the nervous system (93). Studies have shown that dysregulated Eph/ephrin signaling pathway could lead to synaptic deficits associated with AD and suggest that Eph/ephrin signaling pathway could act as a target for new therapeutic opportunities for AD (94, 95). Glycosylation of ephrin has been shown to play an important role in the Eph/ephrin signaling pathway. A study into the interaction between ephrin-A1 and ephA2 demonstrated that deglycosylation of ephrin-A1 decreased its binding affinity to ephA2 and failed to activate the downstream signaling pathways (96). Analysis of Eph/ephrin crystal structures revealed an interaction between the ligand’s carbohydrates and two residues of the ephA2 receptor (94, 96). Our results indicate that decreased ephrin glycosylation in AD may lead to dysregulated Eph/ephrin signaling and thus contribute to AD progression.

A decreased glycosylation, mainly fucosylation, of glycoprotein carnosinase CN1 was also found in AD (Fig. 6*C*). Studies of human plasma carnosinase CN1 have shown there were two potential N-glycosites (Asn322, Asn382) (52), with Asn382 identified in our study. Carnosinase CN1 is a secreted dipeptidases glycoprotein expressed predominantly in the liver and brain and can be selectively secreted by brain cells into CSF, which catalyzes the hydrolysis of the dipeptides carnosine (β-alanyl-L-histidine) (97). Increased levels of CSF carnosinase CN1 have been found in normal aging, while decreased CSF level was found in AD (98). *In vitro* experiments have shown that N-glycosylation is essential for appropriate secretion and enzyme activity (99). Our results indicate that decreased N-glycosylation of carnosinase CN1 may contribute to the decreased level of CSF carnosinase CN1 and may act as a putative marker for AD.

N-glycan structures of alpha-1-antichymotrypsin, ephrin-A3, and carnosinase CN1 were illustrated in Supplemental Figures S8–S10. These glycoprotein candidates with altered glycosylation discussed above are representative examples that may be potentially correlated with AD progression. A complete list of glycoproteins/glycosites with altered glycosylation detected in the present study can be found in Supplemental Tables S12 and 13, with a short description of each glycoprotein’s relevance to AD or ND. Although the comparison is relatively premature given only one pool of each disease and healthy samples with no statistical analysis, the current exploratory glycosylation-based biomarker study focuses on in-depth mapping of the representative and overall glycosylation landscape of CSF proteins in healthy control and AD. Such a comparison will shed light on the glycoproteome profile, dominant glycosylation differences and similarities, and some of the interesting glycoprotein candidates with specific glycosylation pattern alterations in AD. To select potential interesting targets for discussion, we focus on N-glycoprotein/N-glycosite candidates that exhibit at least four glycoform changes for the shared N-glycoproteins/N-glycosites and at least three glycoform changes for the N-glycoproteins/N-glycosites detected only in AD or control. Other factors are also considered including the increased/decreased percentage in terms of the absolute number of detected glycoforms, the reported association with AD or ND, and their roles in CNS biological processes. Nonetheless, this list of N-glycosylated proteins constitute a preliminary exploration and further investigations are needed to narrow down or provide a more complete list of potential interesting glycosylation-based biomarker candidates in AD. Future studies include high-throughput multiplexed quantitative studies using 12-plex DiLeu isobaric tags developed in our lab (100, 101, 102), which would allow us to evaluate individual patient-to-patient variations more efficiently and further validate the results presented in this report.

## Data availability

The LC-MS/MS data have been deposited to the ProteomeXchange Consortium *via* the MassIVE partner repository with the dataset identifier PXD022274.

## Supplemental data

This article contains supplemental data (103, 104, 105, 106, 107, 108, 109, 110, 111, 112, 113, 114, 115, 116, 117, 118, 119, 120, 121, 122, 123, 124, 125, 126, 127, 128, 129, 130, 131, 132, 133, 134, 135, 136, 137, 138, 139, 140, 141, 142, 143, 144, 145, 146, 147, 148, 149, 150, 151, 152, 153).

## Conflict of interest

The authors declare that they have no conflicts of interest with the contents of this article.
