# Supplementary material for: In-depth Site-specific Analysis of N-glycoproteome in Human Cerebrospinal Fluid and Glycosylation Landscape Changes in Alzheimer's Disease
Source: Mol Cell Proteomics. 2021 Apr 20;20:100081. doi: 10.1016/j.mcpro.2021.100081 (PMC8724636; doi:10.1016/j.mcpro.2021.100081)
Supplement: Supplemental Table S12 [file mmc12.docx]

| **Supplemental Table S12-a N-glycoproteins/N-glycosites only detected in control** | | | | | | |
| --- | --- | --- | --- | --- | --- | --- |
| **Uniprot Accession** | **Protein name** | **Glycosylation changes** | **Site** | **No. of glycoforms changes AD VS. control** | **Related to AD/ND** | **Reference** |
| P19022 | Cadherin-2 | 651 | increased total glycosylation | 1 | Synaptic adhesion molecules, these proteins play key roles in formation and maintenance of synapses and regulation of synaptic plasticity, N-glycosylation alters cadherin-mediated intercellular binding kinetics. | (103-104) |
| Q8IUK8 | Cerebellin-2 (Cbln2) | 110 | increased total glycosylation | 3 | belong to the C1q/tumor necrosis factor superfamily, and they are proteins that are essential for the specification, formation, and maintenance of the synapse, Cbln4 enhances inhibitory activity and resistance of neurons to amyloid-β toxicity, Cbln1 is essential for synaptic integrity and plasticity in the cerebellum | (105-106) |
| P16112 | Aggrecan core protein | 126 | increased total glycosylation | 1 | Aggrecan-based perineuronal nets (PNs) of the extracellular matrix have been considered to contribute to neuroprotection, changes in glycosylation with age may influence the ability of aggrecan cleavage | (107-108) |
| Q8N3J6 | Cell adhesion molecule 2 | 211 | increased total glycosylation | 3 | Synaptic adhesion molecules, these proteins play key roles in formation and maintenance of synapses and regulation of synaptic plasticity, N-glycosylation alters cadherin-mediated intercellular binding kinetics. | (103-104) |
| O94856 | Neurofascin | 446,  483,  973 | increased total glycosylation | 1, 1, 1 | Synaptic adhesion molecules, these proteins play key roles in formation and maintenance of synapses and regulation of synaptic plasticity | (103-104) |
| P35052 | Glypican-1 | 116 | increased total glycosylation | 1 | heparan sulfate proteoglycans, glypican-1 interacts with oligomerized or polymerized Abeta, involved in amyloid deposition in senile plaques of AD brain, the N-linked glycans on glypican-1 affect protein expression and heparan sulfate substitution. | (109-110) |
| P52797 | Ephrin-A3 | decreased fuc, sial | 38 | -4, -1 | Glycosylation is needed in the interaction between ephrin and Eph receptors in the Eph/Ephrin signaling pathway, decreased glycosylation of ephrin may lead to deregulated Eph/Ephrin signaling, which further leads to synaptic deficits associated with Alzheimer's disease. | ([91](#_ENREF_91), 93, [95-96](#_ENREF_95)) |

**Supplemental Table S12-b N-glycoproteins/N-glycosites only detected in AD**

| **Uniprot Accession** | **Protein name** | **Glycosylation changes** | **Site** | **No. of glycoforms changes AD VS. control** | **Related to AD/ND** | **Reference** |
| --- | --- | --- | --- | --- | --- | --- |
| Q99523 | Sortilin | 406 | increased total glycosylation | 1 | APOE receptor, implicated in AD | (111) |
| O95180 | Voltage-dependent T-type calcium channel subunit alpha-1H | 271 | increased total glycosylation | 6 | Expressed throughout the nervous system, control cellular excitability and synaptic transmission. Glycosylation of the channel plays important role in its function. Changes in the glycosylation pattern can affect ion channel function, leading to neurological disorders. | (112-113) |
