# Supplementary material for: In-depth Site-specific Analysis of N-glycoproteome in Human Cerebrospinal Fluid and Glycosylation Landscape Changes in Alzheimer's Disease
Source: Mol Cell Proteomics. 2021 Apr 20;20:100081. doi: 10.1016/j.mcpro.2021.100081 (PMC8724636; doi:10.1016/j.mcpro.2021.100081)
Supplement: Supplemental Table S13 [file mmc13.docx]

**Supplemental Table S13 N-glycoproteins/ N-glycosites with an altered glycosylation pattern between control and AD**

| **Uniprot Accession** | **Protein name** | **Glycosylation changes** | **Site** | **No. of glycoforms changes AD VS. control** | **Related to AD/ND** | **Reference** |
| --- | --- | --- | --- | --- | --- | --- |
| P10909 | Clusterin  (apolipoprotein J) | decreased fuc  decreased fuc decreased tetra- decreased sial | 86, 145, 374 374 86 | -4, -8,  -7 -8 -5 | Clusterin levels increased in brain and cerebrospinal fluid in AD, the third most associated AD risk gene, glycosylation alterations for plasma clusterin in AD. | (114) |
| Q96KN2 | Beta-Ala-His  dipeptidase (Carnosinase CN1) | decreased complex, decreased fuc | 382 | -6, -7 | N-glycosylation is essential for appropriate secretion and enzyme activity, decreased levels of carnosinase CN1 in CSF AD, degreased glycosylation may contribute to the decreased secreted CN1 | ([97](#_ENREF_97), 99) |
| P05060 | Secretogranin-1 | decreased fuc | 315 | -7 | Belongs to chromogranin/secretogranin family of neuroendocrine secretory proteins, N-glycosylation may be involved with its sorting and processing, In AD, about 10 to 20% of the amyloid-immunoreactive plaques contained either secretogranin | (115) |
| Q96GW7 | Brevican core protein | decreased complex  fuc, sial, tri- | 337 | -4 -6, -5, -5 | Aggregating extracellular matrix (ECM) proteoglycan that is abundantly expressed in the CNS, defective brevican processing contributes to deficient neural plasticity in AD,  glycosylation is important in its processing | (116-117) |
| P08603 | Complement factor H (CFH) | decreased fuc | 911 | -6 | innate-immune- and inflammation-related regulatory protein in the alternative pathway of the complement system, altered CFH signaling is implicated in the AD process, N-glycosylation play important roles in binding with its natural biological partners | (118) |
| P23142 | Fibulin-1 | decreased fuc, sial | 98 | -5, -4 | Fibulin-1 binds the amino-terminal head of beta-amyloid precursor protein and modulates its physiological function, fibulin-3 requires N-glycosylation to acquire and  maintain a stable, native-like structure. | (119-120) |
| P19652 | Alpha-1-acid glycoprotein 2 | decreased complex,  decreased fuc, bi- | 93 | -7 -5, -4 | CSF biomarker in AD, accurate phase proteins, acute phase negative  modulation of the complement system | (121-122) |
| P13987 | CD59 glycoprotein | decreased complex,  decreased fuc, tri- | 43 | -6 -5, -4 | complement system, CD59 is a cell surface glycophosphoinositol (GPI)-anchored protein that prevents complement membrane attack complex (MAC) assembly, is decreased in AD, glycosylation is involved in reduce the co-stimulation of proliferation and complement inhibitory properties | (123-124) |
| P02787 | Serotransferrin (Tf) | Increased complex  fuc, sial, tri-, pauc decreased fuc, tetra- | 432  630 | +7 +5, +6, +5, +5 -7, -4 | CSF biomarker in AD, Glycosylation of Tf is altered in AD | ([16](#_ENREF_16), 125-126) |
| O95502 | Neuronal pentraxin receptor | decreased complex,  fuc, sial, tetra- | 42 | -5 -5, -4, -5 | CSF biomarker for synaptic dysfunction associated with AD | (127) |
| O60883 | Prosaposin receptor GPR37L1 | decreased complex,  fuc, sial, tri- | 105 | -4, -4, -5, -4 | Orphan G protein-coupled receptors binds to secreted prosaposin, such signaling play its protective actions on neurons and glia, removal of glycosylation causes impairment in the surface expression of GPRs | (128-129) |
| Q8WXD2 | Secretogranin-3 | decreased complex,  fuc, sialyaltion, bi- | 68 | -4, -4, -6, -4 | Belongs to chromogranin/secretogranin family of neuroendocrine secretory proteins, N-glycosylation may be involved with its sorting and processing, In AD, about 10 to 20% of the amyloid-immunoreactive plaques contained either secretoneurin | (130) |
| Q92823 | Neuronal cell adhesion molecule (CD56) | decreased complex,  decreased fuc, bi- decreased sialyaltion | 858 276 | -5, -4, -4 -4 | alterations in synaptic adhesion play key roles in the disruption of neuronal networks in AD, glycosylation affects influence the adhesive function of the molecule. | (103, [131](#_ENREF_131)) |
| P01011 | Alpha-1-antichymotrypsin (ACT) | increased complex,  sial, tri- increased complex, sial | 106 186 | +10, +9, +4 +4 | complement system, altered glycosylation profile of purified plasma ACT from Alzheimer’s disease | (92) |
| O00462 | Beta-mannosidase (MANBA) | increased sial | 763 | +4 | One of lysosomal hydrolases involved in autophagy-lysosomal degradation pathway, maintaining the lysosomal stability, decreased enzyme activity in AD. | (132-133) |
| P00450 | Ceruloplasmin  (caeruloplasmin, ferroxidase, CP) | decreased complex, sial | 358 | -4, -5 | Acute phase protein, Cp is a ferroxidase enzyme and its activity in serum is lowered in AD. It is essential in moving iron out of the reticuloendothelial cells and hepatocytes, is involved in disturbed iron homeostasis in the brain in AD, it's suggested glycosylation pattern of Cp may change in response to AD. | (134-135) |
| P00738 | Haptoglobin (Hpg) | increased complex, sial increased complex, tri- | 207 241 | +5, +5 +4, +4 | Acute phase protein, increased Hpg levels in AD, it participates in antioxidant and anti-inflammatory activities, Haptoglobin Interacts with Apolipoprotein E and Beta-Amyloid and Influences Their Crosstalk in AD | (136-137) |
| P05155 | Plasma protease C1 inhibitor  (C1-Inh, C1 esterase inhibitor) | increased complex, sial, tri- | 238 | +7, +5, +4 | Acute phase protein, inhibition of the complement system, reduced in plasma from AD patients, N-Linked Glycosylation Is Required for C1 Inhibitor-Mediated Protection from Endotoxin Shock in Mice | (138-139) |
| P41222 | Prostaglandin-H2 D-isomerase (PTGDS) | decreased complex,  sial, bi, tri- | 51 | -12, -10, -5, -4 | As endogenous Aβ chaperone, may play an important role in the development of dementia and of Alzheimer's disease, glycosylation forms changes with various brain pathology | (140-141) |
| P01859 | Immunoglobulin heavy constant gamma 2 | Increased complex, tri- | 176 | +5, +4 | Blood plasma IgG Fc glycans are significantly altered in Alzheimer's disease and progressive mild cognitive impairment. | (142) |
| P51693 | Amyloid-like protein 1 (APLP1) | Increased complex, tri-, tetra- | 551 | +11, +4, +4 | APLP-1 is the only member of the APP gene family for which processing can be influenced by N-glycosylation, expressed on cell surface and involved in cell interaction | (143-144) |
| Q9UBP4 | Dickkopf-related protein 3 (DKK3) | Increased complex, tetra- | 106 | +13, +4 | Dickkopf-related protein 3 is a potential Aβ-associated protein in AD | (145) |
| Q96FE7 | Phosphoinositide-3-kinase-interacting protein 1 (PIK3IP1) | Decreased complex | 66 | -4 | PI3K is negatively regulated by PIK3IP1, The PI3K/AKT/GSK-3β pathway has been shown to play a pivotal role in neuroprotection, enhancing cell survival by stimulating cell proliferation and inhibiting apoptosis. This pathway appears to be crucial in AD because it promotes protein hyper-phosphorylation in Tau, glycosylation may be related to ability to its function as an inhibitor of PI3K. | (146-148) |
| P78509 | Reelin | decreased complex | 2961 | -4 | Altered expression and glycosylation patterns of Reelin in cerebrospinal and cortical extracts have been reported in AD. | (149) |
| Q07954 | Prolow-density lipoprotein receptor-related protein 1 (LRP1) | decreased total | N/A | -4 | Same as Apoliprotein D, LRP1 not only regulates the metabolism of amyloid-β peptides (Aβs) in the brain and periphery, but also maintains brain homeostasis, impairment of which likely contributes to AD development in Aβ-independent manners, differential glycosylation as a physiological switch that modulates the diverse biological functions | (150-152) |
| P49908 | Selenoprotein P | decreased total | N/A | -4 | Selenoprotein P (SelP) plays a critical role in neuronal survival and is associated with Alzheimer's pathology. | (153) |
| P55290 | Cadherin-13 | increased total | N/A | +5 | Synaptic adhesion molecules, these proteins play key roles in formation and maintenance of synapses and regulation of synaptic plasticity, N-glycosylation alters cadherin-mediated intercellular binding kinetics. | (103-104) |

Note: fuc: fucosylation, sial: sialylation, complex: complex type, bi, tri, tetra-: bi, tri, tetra-antennary.
