# Supplementary material for: In-depth Site-specific Analysis of N-glycoproteome in Human Cerebrospinal Fluid and Glycosylation Landscape Changes in Alzheimer's Disease
Source: Mol Cell Proteomics. 2021 Apr 20;20:100081. doi: 10.1016/j.mcpro.2021.100081 (PMC8724636; doi:10.1016/j.mcpro.2021.100081)
Supplement: Supporting information [file mmc14.docx]

**Supporting Information**

**In-depth site-specific analysis of N-glycoproteome in human cerebrospinal fluid (CSF) and glycosylation landscape changes in Alzheimer's disease (AD)**

Zhengwei Chen,^1,#^ Qinying Yu, ^2,#^ Qing Yu,^2^ Jillian Johnson,^2^ Richard Shipman,^3^ Xiaofang Zhong,^2^ Junfeng Huang,^2^ Sanjay Asthana,^4^ Cynthia Carlsson,^4^ Ozioma Okonkwo,^4^ Lingjun Li^1,2*^

^1^Department of Chemistry, University of Wisconsin, Madison, WI 53705, USA

^2^School of Pharmacy, University of Wisconsin, Madison, WI 53705, USA

^3^Department of Applied Science, University of Wisconsin-Stout, Menomonie, WI 54751, USA

^4^School of Medicine and Public Health, University of Wisconsin, Madison, Wisconsin 53705, USA.

^#^These authors contributed equally: Zhengwei Chen, Qinying Yu

*Correspondence: Professor Lingjun Li, School of Pharmacy and Department of Chemistry, University of Wisconsin-Madison, 777 Highland Avenue, Madison, Wisconsin 53705-2222

E-mail: lingjun.li@wisc.edu

Fax: +1-608-262-5345

Phone: +1-608-265-8491

**Running title:** Site-specific N-glycoproteomic analysis of CSF in AD

**Supplemental Methods**

**Proof-of-principle experiments using mouse brain tissue**

2 mg of brain tissue extract was subjected to identical trypsin digestion and sequential glycopeptide enrichment as described for CSF glycoproteome analysis. 20% of the total enriched N-glycopeptides was incubated at 37 °C with PNGase F to generate deglycosylated proteins. Both samples were analyzed by LC-MS/MS on the Orbitrap Fusion™ Lumos™ Tribrid™ Mass Spectrometer coupled to a Dionex UPLC system. Raw data was searched through Proteome Discoverer and Byonic, and results were analyzed using the same filtering criteria. All raw data was searched against UniProt *Mus musculus* reviewed database (01.10.2021, 17, 063 sequences), using PTM-centric search engine Byonic (version 2.9.38, Protein Metrics, San Carlos, CA) incorporated in Proteome Discoverer (PD 2.1). Common search parameters were the same as described for CSF glycoproteome analysis. Mammalian N-glycan database embedded in Byonic, which contains 309 glycan entities, was used. Results were analyzed using the same filtering criteria.

**Result cross-validation through pGlyco and MSFragger-Glyco**

CSF raw data was searched through pGlyco and MSFragger using the same common parameters as described for CSF glycoproteome analysis. For pGlyco, the glycopeptide FDR cut-off was 1%. For MSFragger-Glyco, mass offset search was enabled, and filtering was performed with Philosopher (v.3.2.5) to FDR < 1% at PSM level.

**Supplemental Figure Legends**

**Figure S1.** (a) Overlap of identified glycoproteins and deglycosylated proteins in mouse brain tissue. (b) Correlation between glycoprotein intensity with deglycosylated protein intensity.

**Figure S2.** High-pH fractionation comparison between glycoproteomic (a) and proteomic (b) results.

**Figure S3.** The enriched sialylated and non-sialylated glycopeptide percentage comparison between lectin affinity, HILIC and boronic acid methods.

**Figure S4.** (a) Overlap of identified glycoproteins in this study with proteins in Zhong’s global proteomics study. (b) Correlation between glycoprotein intensity with global protein intensity.

**Figure S5.** Overlap of identified N-glycoproteins (a) and N-glycosites (b) with previous human glycoproteomic studies.

**Figure S6.** Overlap of Byonic glycosites with pGlyco (a) and MSFragger-Glyco (b).

**Figure S7.** A two-dimensional plot depicting the changes in the number of fucosylated (a) and sialylated (b) glycoforms on each site (AD vs. Control) as a function of the number of total identified glycoforms in AD.

**Figure S8.** Identified N-glycan structures from alpha-1-antichymotrypsin.

**Figure S9.** Identified N-glycan structures from ephrin-A3.

**Figure S10.** Identified N-glycan structures from carnosinase CN1.

**Supplemental Table Legends**

**Supplemental Table S1.** Subjects’ information of 16 healthy control and 16 AD patients.

**Supplemental Table S2.** N-Glycoproteins and deglycosylated proteins identified from mouse brain tissue.

**Supplemental Table S3.** N-glycopeptides identified using different enrichment strategies.

**Supplemental Table S4.** N-glycopeptides identified, N-glycoforms identified, and the shared N-glycosites and N-glycoproteins in control and AD.

**Supplemental Table S5.** Comparison with previous human CSF proteome and N-glycoproteome studies.

**Supplemental Table S6.** Glycosite cross-validation through pGlyco and MSFragger-Glyco.

**Supplemental Table S7.** Gene ontology analysis of the N-glycoproteins identified in CSF from healthy control.

**Supplemental Table S8.** 10 N-glycoproteins with more than 5 N-glycosites detected.

**Supplemental Table S9.** Heat map of delta number of detected glycoforms (changes in the number of AD-Control) on the shared N-glycosites detected from control and AD.

**Supplemental Table S10.** N-glycoproteins/N-glycosites detected only in AD.

**Supplemental Table S11.** N-glycoproteins/N-glycosites detected only in control.

**Supplemental Table S12.** Interesting N-glycoproteins/ N-glycosites detected only in control or AD, and their relations to AD or neurodegenerative disease (ND).

**Supplemental Table S13.** Interesting N-glycoproteins/ N-glycosites with an altered glycosylation pattern shared between control and AD, and their relations to AD or neurodegenerative disease (ND).

Please note that Supplemental Tables S1-S11 are provided as separate Excel files, whereas Supplemental Tables S12-S13 are provided as PDF files.

**Supplemental Figures**

**Figure S1. Chen et al.**

**
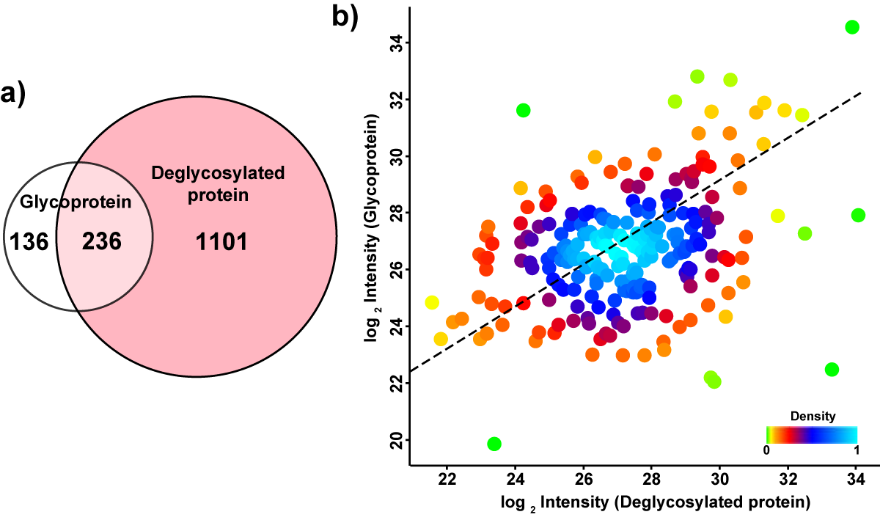
**

**Figure S2. Chen et al.**

**
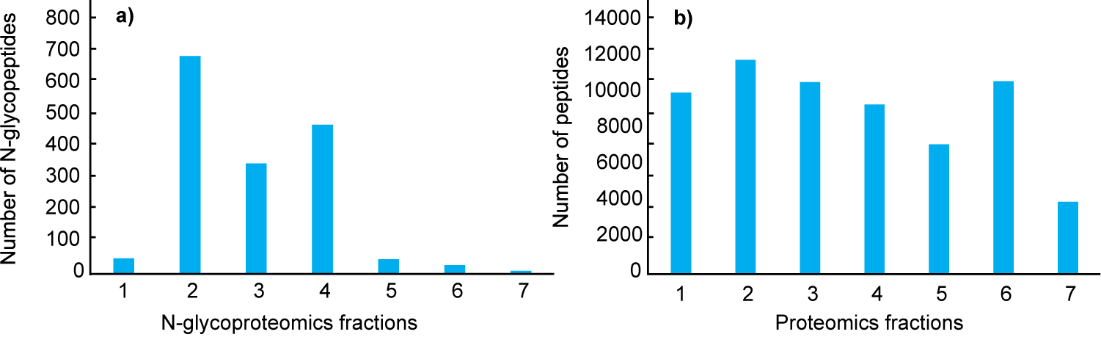
**

**Figure S3. Chen et al.**

**
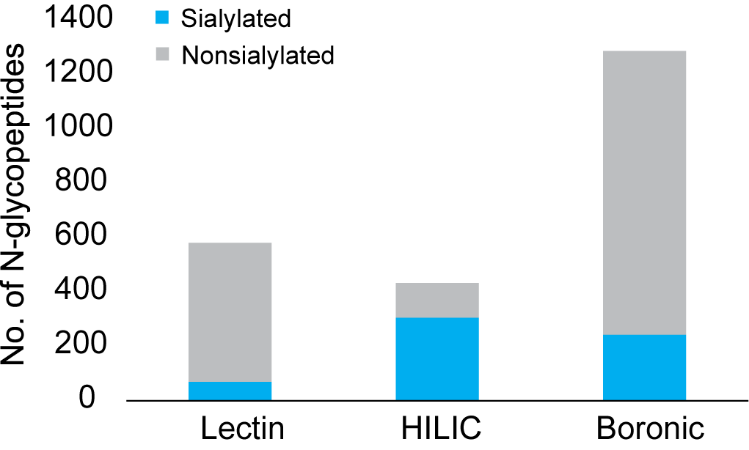
**

**Figure S4. Chen et al.**

**
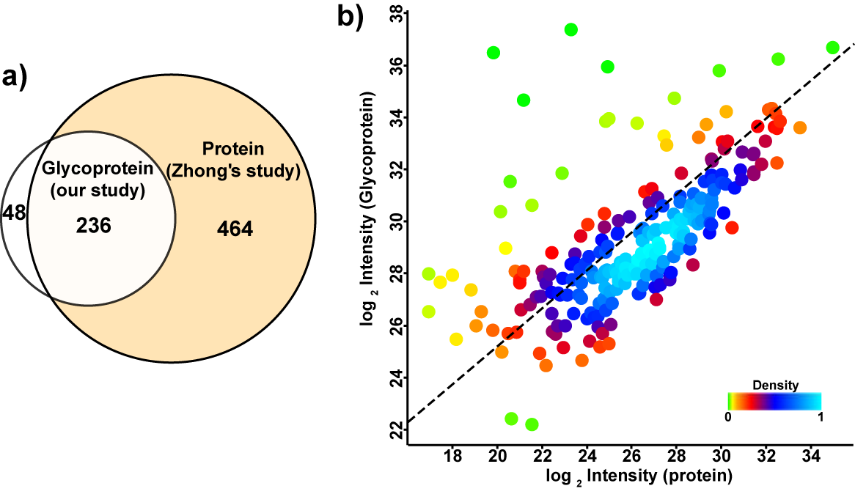
**

**Figure S5. Chen et al.**

**
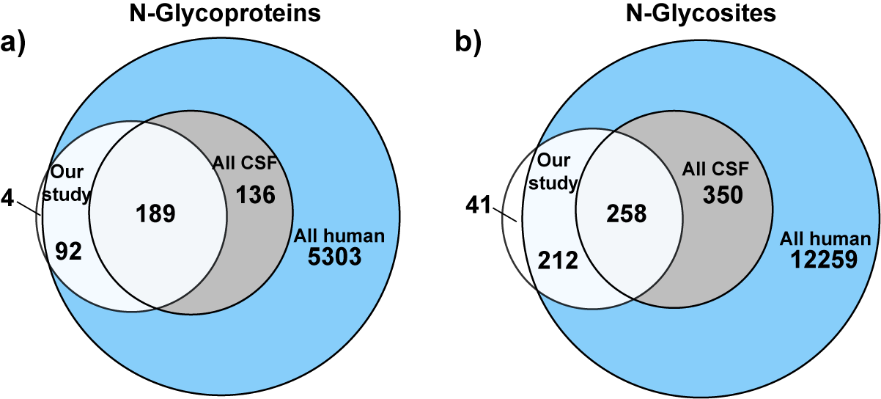
**

**Figure S6. Chen et al.**

**
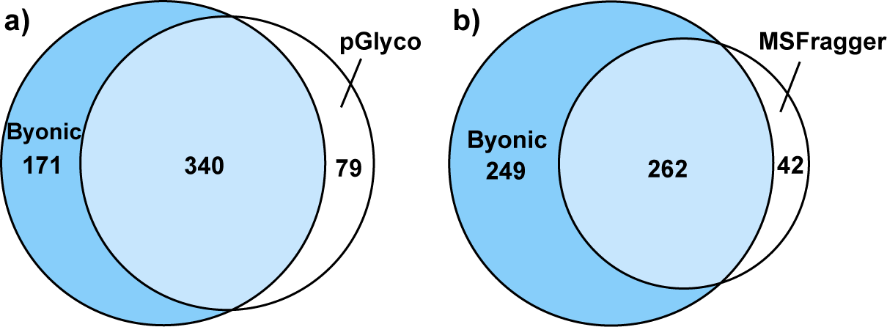
**

**Figure S7. Chen et al.**

**
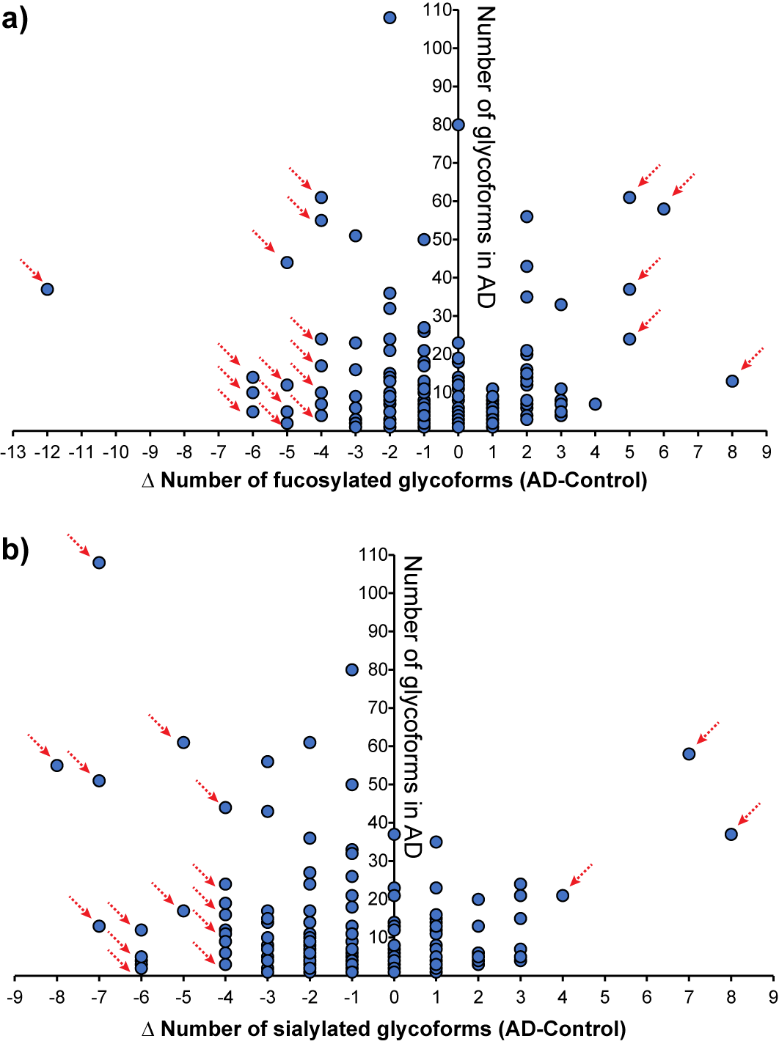
**

**Figure S8. Chen et al.**


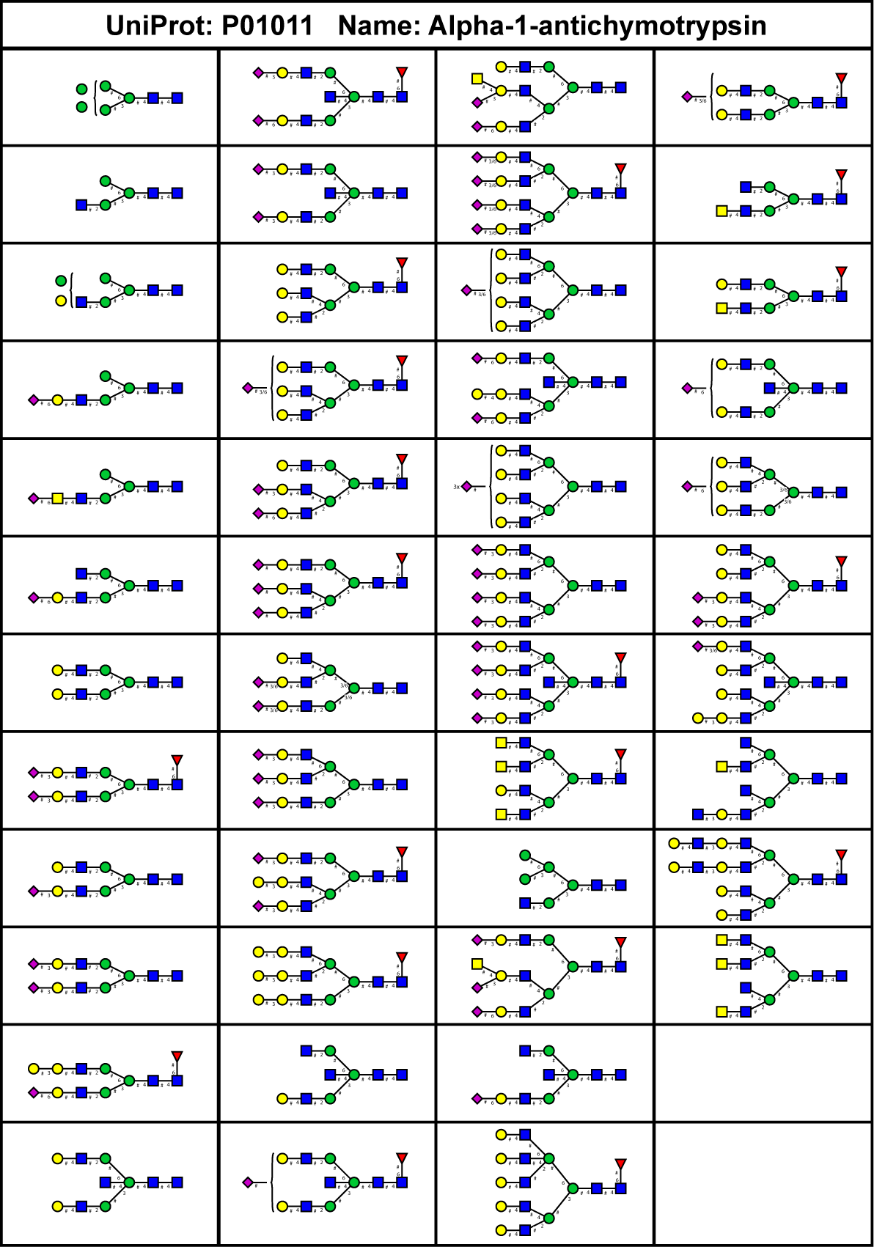


**Figure S9. Chen et al.**


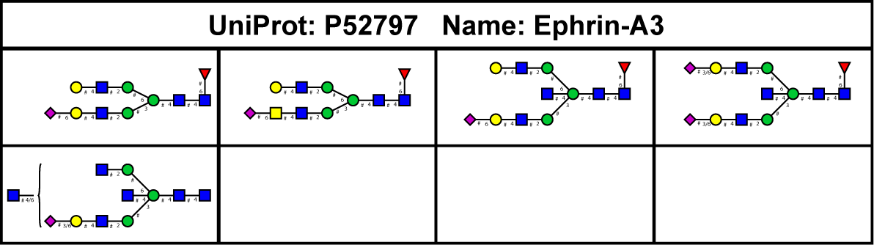


**Figure S10. Chen et al.**

**
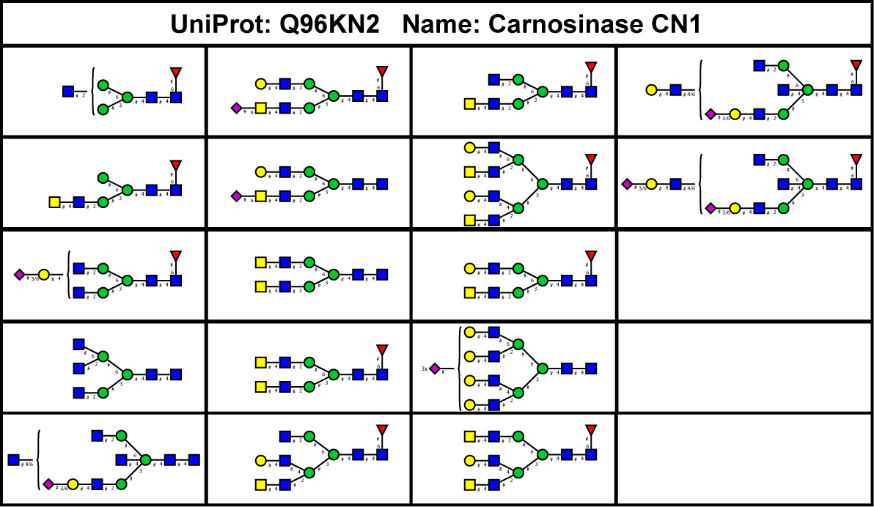
**
